# Review of the impact of heat stress on reproductive performance of sheep

**DOI:** 10.1186/s40104-020-00537-z

**Published:** 2021-02-15

**Authors:** William H. E. J. van Wettere, Karen L. Kind, Kathryn L. Gatford, Alyce M. Swinbourne, Stephan T. Leu, Peter T. Hayman, Jennifer M. Kelly, Alice C. Weaver, David O. Kleemann, Simon K. Walker

**Affiliations:** 1 The University of Adelaide, School of Animal and Veterinary Sciences, Davies Livestock Research Centre, Roseworthy Campus, Mudla Wirra Rd, Roseworthy, South Australia 5371 Australia; 2 The University of Adelaide, Robinson Research Institute, Adelaide Medical School, North Terrace, Adelaide, South Australia 5000 Australia; 3 The University of Adelaide, School of Agriculture, Food and Wine, Waite Research Institute, Urrbrae, South Australia 5064 Australia; 4 South Australian Research and Development Institute, Primary Industries and Regions SA, Government of South Australia, Climate Applications, Waite Research Precinct, Urrbrae, South Australia 5064 Australia; 5 South Australian Research and Development Institute, Primary Industries and Regions SA, Reproductive Biology, Livestock Sciences, Turretfield Research Centre, 129 Holland Rd, Rosedale, South Australia 5350 Australia

**Keywords:** Fertility, Heat stress, Oogenesis, Pregnancy, Sheep, Spermatogenesis

## Abstract

Heat stress significantly impairs reproduction of sheep, and under current climatic conditions is a significant risk to the efficiency of the meat and wool production, with the impact increasing as global temperatures rise. Evidence from field studies and studies conducted using environmental chambers demonstrate the effects of hot temperatures (≥ 32 °C) on components of ewe fertility (oestrus, fertilisation, embryo survival and lambing) are most destructive when experienced from 5 d before until 5 d after oestrus. Temperature controlled studies also demonstrate that ram fertility, as measured by rates of fertilisation and embryo survival, is reduced when mating occurs during the period 14 to 50 d post-heating. However, the contribution of the ram to heat induced reductions in flock fertility is difficult to determine accurately. Based primarily on temperature controlled studies, it is clear that sustained exposure to high temperatures (≥ 32 °C) during pregnancy reduces lamb birthweight and will, therefore, decrease lamb survival under field conditions. It is concluded that both ewe and ram reproduction is affected by relatively modest levels of heat stress (≥ 32 °C) and this is a concern given that a significant proportion of the global sheep population experiences heat stress of this magnitude around mating and during pregnancy. Despite this, strategies to limit the impacts of the climate on the homeothermy, behaviour, resource use and reproduction of extensively grazed sheep are limited, and there is an urgency to improve knowledge and to develop husbandry practices to limit these impacts.

## Introduction

The thermal environment is the largest single stressor affecting the efficiency of animal production systems, impacting development, growth and reproduction of all animals [1]. Thermal impacts on sheep performance and wellbeing are most profound when temperatures fall below 12 °C (lower critical temperature) or rise above 25 to 31 °C (upper critical temperature), when thermoregulatory mechanisms are seriously challenged and the ability of sheep to maintain homeothermy is reduced. However, well before these temperatures are reached, the physiological and behavioural adaptations that allow sheep to maintain homeothermy negatively impact their growth, welfare and reproduction. These adaptations include increased heat dissipation and decreased metabolic heat production, through peripheral vasodilation and reduced food intake. Prolonged exposure to thermal stress also induces endocrine adaptations which further decrease metabolic rate and promote heat dissipation [2–5]. Behavioural responses to thermal stress include a reduction or temporal shift in activity to cooler parts of the day, and increased use of shade [5]. These changes may further impact production and reproduction by reducing foraging time and/or increasing distances sheep travel to feed and water.

Importantly, temperatures are projected to increase globally and sheep will therefore be exposed to higher temperatures more frequently and for longer periods. In light of this, heat stress has been identified as one of the key vulnerabilities facing the sheep meat and wool production industries [6–8]. Based on observed and projected increases in temperature associated with anthropogenic climate change, the consequences of exposure of sheep to thermal stress will become increasingly serious. This review therefore describes the effects of heat stress on both ewes and rams, including effects around mating on fertility, oogenesis and spermatogenesis, and effects during pregnancy and lactation on pregnancy outcomes, milk production and progeny wellbeing. We also discuss relevant underlying physiological mechanisms and identify gaps in current knowledge relevant to sheep production in grazing environments of high ambient temperature.

## Impact of heat stress on ewe fertility

Evidence from a small number of extensive Australian and Spanish field studies demonstrates the significant, negative impact that heat stress has on the reproductive performance of commercially managed flocks. In two Australian studies, each utilising data from more than 50 Merino flocks, ewe fertility (the number of ewes lambing per 100 ewes mated) and lambing rate (number of lambs born per 100 ewes mated) correlated negatively with the number of days per week during the mating period when ambient temperatures were ≥ 32.0 °C [9, 10]. For each additional day ≥32 °C during the week of mating, ewe fertility and lambing rate decreased by 2.7% and 3.5% respectively [9, 10]. The results of these studies indicate that high temperatures during the mating period adversely impact fertilisation, embryo survival and, consequently, pregnancy rates. In partial support, data generated from over 150 Spanish flocks demonstrate a significant decline in pregnancy rates when maximum daily temperatures were ≥ 30 °C for 2 d before artificial insemination [11, 12]. Lindsay et al. [9] also reported a negative correlation between the number of lambs born per 100 ewes joined and daily maximum temperatures in the 3 weeks after mating indicating that heat stress post-mating continues to impair ewe fertility. Using equations developed by Lindsay et al. [9], in conjunction with Australian Gridded Climate Data [13] we estimated that 2.1 million potential lambs are lost due to heat stress (days > 32 °C) around mating, costing the Australian sheep industry $97 million annually [14].

**Fig. 1:**
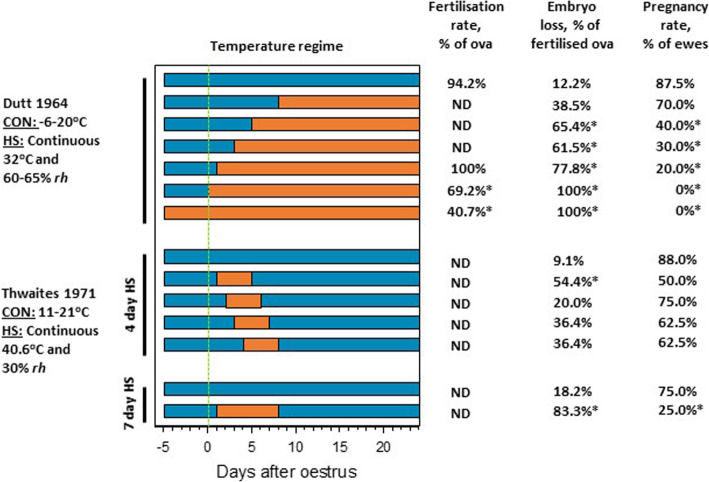
Impacts of heat stress of ewes around mating on fertilisation rate, embryo loss (encompassing oocyte loss) and pregnancy rate. Timing of normothermic (blue) and hyperthermic (orange) temperatures is shown for each treatment. CON, Control; HS, Heat Stress; *rh*, relative humidity; ND, Not determined. *indicates significant difference from control group. Data are from [15, 18]

Under temperature controlled (hot-room) conditions, exposing ewes to high temperatures (32 °C) either shortly before the onset of oestrus or during early oestrus reduced fertilisation rates [15–17]. Applying heat stress from d − 5, d 0 or d 1 relative to the day of onset of oestrus (d 0), dramatically reduced pregnancy rates (Fig. 1; [15, 17, 18]). Pregnancy rates were also reduced when heat stress started 3 or 5 d after mating but were unaffected when heat stress was applied from d 8 (Fig. 1). Overall, meta-analysis indicates that heat-stressed ewes are 2.4 times less likely to get pregnant than thermo-neutral ewes [19]. Based on data summarised in Fig. 1 and evidence from field studies described above [9–12, 20] we conclude that the reproductive performance of the ewe is most affected by heat stress in the week prior to oestrus, during oestrus and then for the ensuing 5 d. It is also of note that shearing decreases the negative impact of heat stress during the peri-oestrus period on pregnancy rates [17], and that fertilisation rates are not reduced by heat stress when lower temperatures are provided overnight [21, 22].

### Mechanisms for effects of heat stress around mating on ewe fertility

#### Oestrous cycle

Consensus from the literature is that heat stress reduces the duration of behavioural oestrus, the incidence of oestrus and the length of the cycle. Although the systematic review of data [19] indicates a decrease in the length of behavioural oestrus by an average of 7.1 h, the timing of heat stress relative to the day of oestrus (d 0) is pivotal. The duration of oestrus was 8.4 h and 5.5 h shorter when constant heat stress was applied either d − 5 to d 0 or d − 3 to d 0 respectively, with the intensity of oestrus behaviours also reduced [23]. However, the duration of oestrus was unaffected by exposure to constant heat stress between d − 7 to d − 4 [23], or when heat stress was applied only diurnally between d − 5 to d 0 [21, 24]. In contrast, in Indian native breeds, the duration of oestrus was 6 h and 8 h shorter, respectively, when ewes experienced diurnal heat stress from 28 or 35 d prior to oestrus [22, 24].

On the other hand, the incidence of oestrus in Merino ewes exposed to constant heat stress from d − 5 to d 0, was reduced by 25% to 30% [23, 25]. However, this parameter was unaffected when constant heat stress was applied from d − 7 to d − 4 or d − 3 to d 0 [25], or when ewes experienced only diurnal heat stress for the 5 d (Merino [20]) or the 35 d (Malpura [23]) prior to oestrus. There is also no evidence that the incidence of oestrus in the field is affected by heat stress [10, 26], likely reflecting homeothermic responses to cooler night-time temperatures. However, oestrous activity can be irregular in Merino ewes in hotter regions of Australia [27–29], and ewe and ram behaviour can be similarly impaired during heat stress [30].

In comparison with both the duration and incidence of oestrus, the effect of heat stress on the length of the oestrous cycle appears more benign. Mean cycle length was increased by between 0.6 and 1.7 d, when heat stress was applied from d − 5 to d 0 [17, 23, 25]. However, cycle length was unaffected when Merino ewes experienced constant heat stress from either d − 7 to d − 4 or d − 3 to d 0 [25], or when Malpura ewes experienced diurnal heat stress for the 35 d preceding oestrus [24].

The underlying mechanisms for effects of heat stress on the oestrous cycle are poorly understood, although changes in circulating hormone concentrations induced by heat stress during the final stages of follicle growth and differentiation may be involved. Endocrine changes include reduced luteinising hormone (LH) secretion [31, 32] with fewer LH pulses [33], in turn reducing stimulation of follicle growth and development. Compared to control groups, heat stress reduced oestradiol concentrations and aromatase activity in the dominant follicles of ewes [33, 34] and circulating oestradiol concentrations in cows [35–38], which might impact oestrous incidence and duration. Circulating progesterone during the luteal phase is higher in heat-stressed compared to control ewes [23, 39–41], suggesting increased progesterone production and/or decreased clearance, which may subsequently affect the timing and progression of pre-ovulatory follicle growth.

#### Follicle growth and ovulation rate

Most of what is known of the effects of heat stress on follicle dynamics is from studies in dairy cows exposed to seasonal variations in ambient temperature. These studies indicate multiple effects of seasonal heat stress on follicle growth and development [34, 37, 42–45]. These effects include more large follicles in the first follicle wave, an increase in the number of small and medium follicles, suppression of the dominant follicle of both the first and second waves, an earlier emergence of the dominant follicle of the second wave, as well as a decline in follicle aromatase activity and plasma oestradiol concentrations. These changes likely reflect altered function of follicular cells and/or changes in the secretion of gonadotrophins [45]. In the field, there are associated reductions in the rates of embryo survival and cow fertility [46–48]; however, the extent to which these findings also apply to sheep is yet unknown.

Cattle studies also demonstrate adverse effects of heat stress on follicle growth and function. *In vivo* heat stress applied for 20 to 26 d reduced *in vitro* steroid production from follicle cells [49] whilst 12 h of *in vitro* heat stress induced early activation of primordial follicles, reduced the rate of oocyte nuclear maturation and disrupted steroid production [50]. Comparable impaired nuclear maturation of oocytes *in vitro* has been reported [51–53]. One study [33] examined the effects of acute heat stress on follicle dynamics during the synchronised oestrous cycle in the goat. Heat stress (36 °C, 70% relative humidity; *rh*) applied from 48 h before until the time of the last prostaglandin injection (i.e. ~ 24 to 72 h before onset of oestrus) delayed recruitment of dominant follicles by 24 h, and tended to delay the time of ovulation. These changes in follicle dynamics occurred in conjunction with lower concentrations of oestradiol in plasma and follicular fluid, and lower aromatase activity and LH receptor expression in follicles.

Under controlled conditions, heat stress prior to oestrus induces non-significant reductions in ovulation rate in sheep [15, 17, 21, 54, 55], a finding that is surprising given the effects of hyperthermia on follicle dynamics in cows. In the field, the effect of heat stress on ovulation rate is inconsistent [9, 10], whilst in superovulated ewes there was no significant effect [22].

#### Oocyte quality

In several studies, heat stress applied before and/or during behavioural oestrus induced a marked increase in the incidence of cytoplasmic vacuoles, cytoplasmic globules, ruptured oolemma and cracked zona pellucida [15–17, 56]. Unlike fertilisation rate, the incidence of abnormal ova remained high when heat stress was interspersed with cooler temperatures [41]. One limitation of these studies is that the ova were approximately 3 or 7 d of age at the time of collection.

Aberrant maturation of oocytes is the most likely cause of fertilisation failure following hyperthermia. Heat stress *in vitro* impaired nuclear and cytoplasmic maturation of bovine oocytes [57–60], and significantly decreased the percentage of ovine oocytes progressing past M1 [61, 62]. This decrease is likely to be a consequence of elevated concentrations of reactive oxygen species (ROS) in embryos and/or oviducts [63–65]. A similar increase occurs in ovine oocyte – cumulus complexes when matured *in vitro* at 42 °C compared with 39 °C [66]. Further evidence that elevated ROS levels contribute to heat-induced impairment of oocyte maturation comes from the heat-associated reduction in intracellular glutathione concentrations [67] and the ameliorating effects of antioxidant treatments on meiotic maturation and developmental competence of heat-exposed oocytes [63, 65, 66]. Interestingly, in the study of Ahmadi et al. [61], oocytes collected in summer were more resistant to thermal stress during *in vitro* maturation compared to oocytes collected in winter; however, how *in vivo* heat exposure might protect oocytes from subsequent *in vitro* heat stress is unknown.

#### Ovum wastage and early embryo loss

It is clear from field studies [9, 10] and studies conducted using environmental chambers that heat stress in the peri-oestrous period increases fertilisation failure and early embryo loss. The proportion of ewes returning to oestrus, indicative of fertilisation failure, was positively associated with the number of days ≥32 °C during the mating period, increasing by 3.44% for every additional day ≥32 °C [10]. High ambient temperatures after mating also appear to increase embryo loss [9]. Compared to control ewes, embryo mortality was markedly higher as a result of heat exposure (32 °C) from 5 d before oestrus, with all embryos being lost when heat stress commenced on the day of oestrus (Fig. 1; [15, 18]). Similarly, exposing maiden and mature ewes to heat stress from d − 2 to d 0 of oestrus increased embryo mortality by 71.4% and 63.6%, respectively [68]. A marked reduction in fertilisation rate occurred in ewes exposed to high temperatures from d − 5 to d 0 of oestrus, inclusive [15, 16].

Although significant ovum/embryo wastage also occurs when heat stress is applied after oestrus, the severity declines as the time between oestrus and exposure increases (Fig. 1). Four days of heat stress after mating increased ovum/embryo wastage when heat stress occurred the day after oestrus (Fig. 1). Similarly, the impact of heat stress on wastage continued until d 24 post-mating which decreased progressively as the number of days between mating and the start of heat stress increased (Fig. 1). Shearing ewes also reduced the extent to which ovum/embryo wastage increased in response to continuous heat stress for the first 15 d after mating [68]. When heat stress of Merino and Southdown ewes was sustained from mating through the first 20 d of pregnancy, 75% of ovum/embryo wastage occurred prior to d 12 [69], the time of maternal recognition of pregnancy. Importantly, when partial diurnal relief of heat intensity was provided, embryo loss was not elevated [68].

Meta-analyses [19] indicate that ewes exposed to short term heat stress are 12.4 times more likely to experience embryo mortality or have unfertilised ova compared to control ewes, which increases to 26.3 times more likely with moderate periods of heat stress. Further, based on the studies reviewed, it is evident the impacts of heat stress on embryo mortality are most severe during the period immediately preceding oestrus, with the day of oestrus being a critical window. Although it was concluded [67] that appreciable embryo mortality in the field, as a consequence of heat stress, is likely only under severe heat wave conditions, evidence of embryo loss in commercial flocks has been provided [9]. Specifically, these authors [9] report a negative correlation between lambing rates and daily maximum temperatures in the 3 weeks following mating.

It is concluded that heat stress compromises oocyte maturation resulting in reduced rates of fertilisation with associated, independent effects on embryo wastage. In this context, it is noteworthy that immature oocytes can be fertilised and undergo cleavage without developing to the blastocyst stage [70]. Additionally, ROS production in the oviducts [63] is likely to increase embryo mortality, consistent with the observation that the effects of heat stress are greatest in the days immediately following oestrus [15, 16, 18, 71]. Further, heat stress adversely affects the oviduct environment as suggested by reduced sperm numbers and increased proportions of sperm-free oocytes in the oviducts of ewes exposed to heat stress [17].

## Effects of heat stress on ram fertility

Maintenance of testicular temperature below core body temperature is required for optimal production of motile and morphologically normal ovine spermatozoa [72]. Intra-testicular temperatures of 33 to 35 °C are reported in rams at ambient temperatures of 20 to 30 °C, representing a rectal-testicular temperature gradient of between 4 and 6 °C [73–76]. Numerous experimental studies using either climate-controlled rooms, or localised scrotal heating, report adverse effects of heat stress on spermatogenesis and semen quality in rams [72, 77]. However, few studies have systematically assessed temperature effects on semen quality under field conditions.

In addition to potential temperature effects, seasonal changes in testicular function can also be influenced by photoperiod and nutrition [78]. Some variation in semen quality between seasons is indicated in Egyptian breeds and conditions [79]. No consistent trend in semen characteristics were observed in Merino rams studied for 18 months [80]. However, this varied between rams, with 4 of 11 rams showing seminal degeneration during summer [80]. The field studies of Lindsay et al. [9] and Kleemann and Walker [10] observed no relationship between mean maximum temperature in the 3 weeks preceding mating and lambing outcomes, suggesting a limited contribution of heat-induced effects on semen quality to pregnancy outcomes. However, variation between rams in susceptibility to heat stress could limit assessment of male contributions to heat-induced effects in flock mating studies, if less-affected rams fertilise the majority of ewes.

### Effects of heat stress on spermatogenesis and semen quality

Experimental studies provide clear evidence for an effect of heat exposure on semen quality. Increased percentages of morphologically abnormal sperm are reported following various heat exposures, including scrotal insulation for 30 h [81], and hot-room exposure at 41 °C for 9 or 13.5 h [82], 32 °C for 4 d [83] or 40.5 °C for 8 h/d for 2 to 5 d [84–86]. Abnormalities become evident from around 9 d post-heating, peaking at 18 to 24 d, and persisting until 30 to 35 d after treatment. Reduced percentages of motile sperm were also detected from approximately 14 to 24 d following heat stress. For example, semen samples collected following 21 d of 16 h/d scrotal insulation contained approximately 20% motile sperm [77, 87]. Similarly, heating to 32 °C, 65% *rh* for 4 days reduced motile sperm from 80% to < 10% 2 weeks after treatment [83]. Sustained effects were also reported when Merino rams were exposed for 3 d at approximately 35 °C, reducing motility from 15 to 35 d post-heating [88]. While increased percentages of abnormal and immotile sperm are consistently reported following heat exposure, effects on sperm concentration were variable, and can be influenced by the frequency of semen collection. For example, decreased sperm numbers were reported from 25 to 30 d after 2 h scrotal heating to 41 °C, with low numbers maintained to d 50 to 60, while heating to 39 °C for 4 h had limited effects [89]. Heat exposure in environmental chambers (40.5 °C, 45% *rh*, 8 h/d) for 2 to 5 d also reduced sperm density across 48 d post-heating; however, this decrease was significant only in rams exposed for 5 d (↓ 26%) [85].

**Fig. 2:**
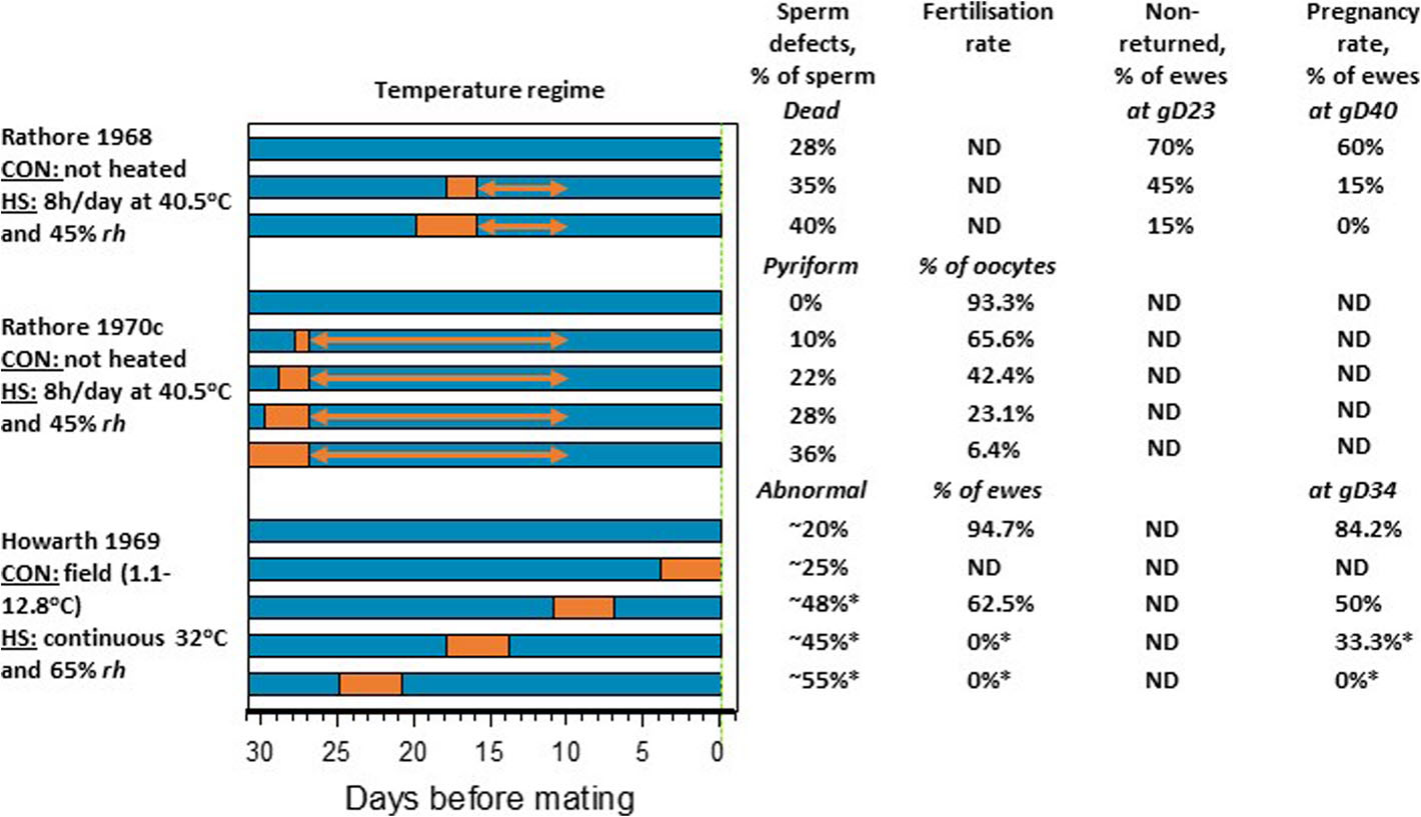
Impacts of heat stress of rams before mating on sperm morphology, and fertilisation and pregnancy and lambing rates after mating to non-heat stressed ewes. Timing of normothermic (blue) and hyperthermic (orange) temperatures is shown for each treatment. CON, Control; HS, Heat stress; *rh*, relative humidity; ND, Not determined; *indicates significant difference from control group; arrows indicate range of timing of heat stress prior to semen collection for mating (data derived from [83, 84, 86, 92])

Studies have also considered the effects of heat exposure of rams on subsequent fertilisation and pregnancy rates, with effects reported following scrotal heating for 1.5 to 2 h [89–91], and whole body heat exposure in environmental chambers at 32 °C for 4 d [83] or 40.5 °C for 8 h/d for 2 to 4 d [84, 92, 93] (Fig. 2). Negative impacts of exposing rams to 4 d of heat stress (32 °C, 65% *rh*) on fertilisation rates and embryonic survival become evident 2 weeks after heating, with no fertilised embryos detected when mating occurred 3 weeks post-heating (Howarth, 1969; Fig. 2). Fertilisation and pregnancy rates decreased as the number of hot (40.5 °C, 45% *rh*) days experienced by rams 10 to 16 [93] or 10 to 27 [92] d prior to mating increase (Fig. 2). This reduction in ram fertility reflects the increase in abnormal spermatozoa which occurred as heat duration increased [84–86, 93]. Lower pregnancy rates at d 40, compared with d 23, in ewes mated to rams exposed to heat 10 to 16 d prior to mating (40.5 °C, 8 h/d, 2 or 4 d) also suggests that, in addition to reduced fertilisation, embryo or early fetal loss may contribute to reduced pregnancy rates from heat-exposed rams ([92]; Fig. 2). The potential for embryonic loss following fertilisation with heat-affected sperm is supported by studies in mice, where exposing males to whole body or scrotal heating reduced embryo developmental competence [94–96].

### Mechanisms of heat stress effects on spermatogenesis and semen quality

Physiological mechanisms contributing to thermoregulation of the ovine testes are well-described. The pampiniform plexus provides countercurrent heat exchange between arterial blood entering the scrotum and cooler venous blood exiting, under normothermic conditions [73]. Scrotal responses to temperature change, including discharge of scrotal sweat glands and variation in scrotal surface area and degree of contraction towards the body, mediated by tunica dartos and cremaster muscles, have a primary role in testes thermoregulation [97, 98]. Heating the scrotum above 36 °C also increases respiration rate of the woolled ram [99–101] and tunica dartos, sweat gland and respiratory responses to temperature are mediated through cutaneous scrotal thermoreceptors [98, 100, 102, 103]. When the capacity of these thermoregulatory mechanisms is exceeded, significant increases in testicular temperature can occur. Elevations in scrotal or testicular temperature by approximately 6 to 8 °C are reported in scrotal heating studies [91, 102]. Similarly, exposure of Merino rams to 45 °C for 3 h elevated scrotal and testicular temperatures by approximately 4 to 5 °C, while variation in rectal temperature was less than 1 °C [104]. However, effects of elevated ambient temperatures on scrotal or testicular thermoregulation under field conditions have been little studied.

Scrotal temperature during heating has also been shown to correlate with semen quality measures. Testes temperature, during 2 h scrotal heating, correlated negatively with pregnancy rates using semen collected 14 to 21 d later [91]. Maximum subcutaneous scrotal temperature in rams exposed to 41 °C for 4 to 13.5 h also correlated negatively with a mean semen composite score, based on motility, morphology and concentration measures [82]. Similarly, maximum scrotal temperature in rams exposed to two 6 h periods of 40.5 °C correlated positively with semen damage score, across 13 to 52 d post-heating [105]. These authors observed variation between rams, with some showing significant seminal degeneration following heat exposure; however, limited effects in others, possibly because they were able to maintain scrotal temperatures during heating 2 to 3 °C lower than affected rams [105]. Others also reported variability between individual rams in the responses to heat stress [77, 82]. Similarly, individual variation in scrotal thermoregulatory ability is also evident in Wagyu bulls [106]. In Merinos, early studies demonstrated variation in scrotal thermoregulatory ability in rams selected for high or low skin fold scores, including differences in testicular blood flow, scrotal surface area, and sweat gland size and density [75, 88, 90, 104, 107], with high skin fold rams having greater susceptibility to heat-induced effects. Testicular thermoregulatory capacity also varies between *Bos indicus* and *B. taurus* bulls, potentially related to differences in scrotal and testicular morphology [108]. Variation between sheep breeds in susceptibility to heat-induced effects on semen quality has also been reported [109]. Understanding the extent to which scrotal thermoregulatory capacity varies between rams within breeds, and between breeds, and the mechanisms underlying variation in the ability to regulate testicular temperature, could be beneficial when considering future strategies for management under heat stress conditions.

The effects of increased testicular temperature on semen quality can include germ cell apoptosis, DNA damage, and perturbation of sperm maturation, including induction of structural and functional abnormalities [72, 84–86, 110, 111]. Effects vary with cell stage, with pachytene spermatocytes and round spermatids identified as particularly susceptible to heat-induced apoptosis [110, 112, 113], while heat stress during later stages of spermatogenesis and epididymal maturation is associated with the induction of structural and functional abnormalities [84–86]. Spermatozoa take up to 14 d to transit the ovine epididymis [114], and therefore, abnormalities appearing 9 to 10 d post-heating may originate in the epididymis. The continued presence of abnormalities until 30 d after heating suggests they can also be induced during spermatogenesis [84–86]. Hypoxia is suggested as a mechanism through which hyperthermia induces germ cell damage [72, 74]. However, while an early study [74] reported no increase in testicular blood flow when ram testes were heated, a recent study demonstrated increased testicular blood flow and oxygen extraction in anaesthetised rams with sequential increases in testicular temperature to 40 °C [115]. Spermatozoa are vulnerable to oxidative stress-induced damage, including membrane lipid peroxidation and DNA damage [116], and scrotal heat stress in rodents induced expression of oxidative stress markers [113, 117]. Although testis function can be affected by nutrition [118], thermal stress is a more significant stressor for sperm production. In Malpura rams, heat stress (42 °C, 55% *rh*, 6 h/d for 45 d), reduced semen mass motility and concentration, while nutritional restriction to 30% *ad libitum* intake had minimal effects on these measures [119]. Semen quality in rams exposed to both heat and nutritional stress did not differ from those exposed to heat stress alone [119].

In summary, despite considerable experimental evidence for effects of heat stress on spermatogenesis and semen quality, the contribution of the ram to heat-induced reductions in flock fertility remains equivocal. This likely reflects the significant delay (2 to 4 weeks) between exposure to heat stress and reductions in ram fertility, as well as the sensitivity of spermatogenesis to relatively short (1 to 4 d) periods of high temperature. Further, rams vary in their susceptibility to heat stress and ability to maintain scrotal temperature, and in flock mating studies where teams of rams were used, it is probable the majority of ewes are fertilised by the less effected rams.

## Impact of heat stress during pregnancy

### Birthweight and fetal growth

**Fig. 3:**
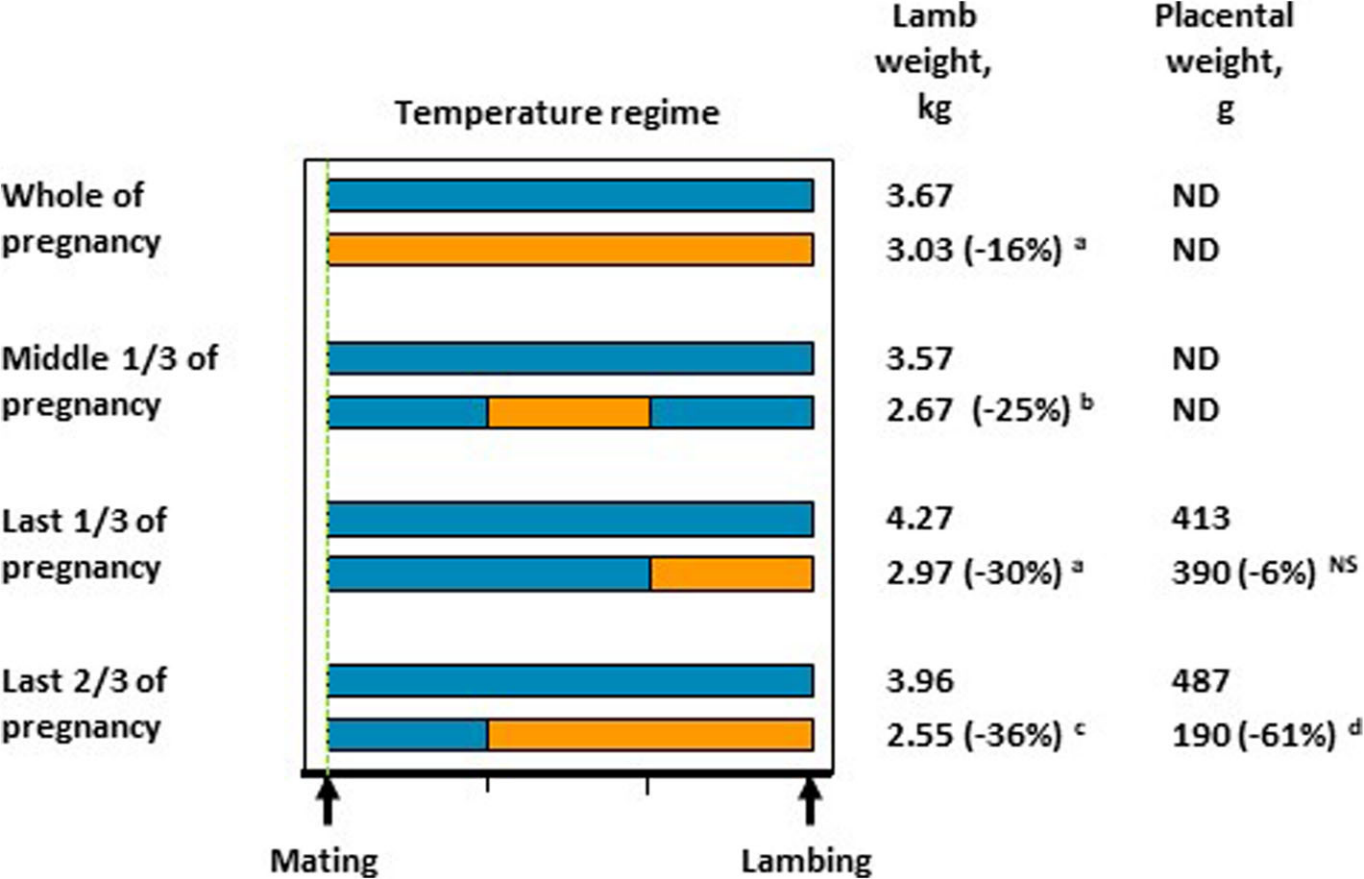
Effect of sustained exposure to elevated ambient temperature during pregnancy on lamb and placental weight around birth. Weighted means for lamb weights and placenta weights at term were calculated from studies in which hyperthermic conditions were induced using approximate circadian rhythms, and measures were taken from either new born lambs, or fetuses within 15 d of predicted full term. Lamb birth weight data were derived from the following 15 papers: [20, 26, 126–136]. Placental weight data were derived from the following 5 papers: [126, 130–132, 135]

Sustained exposure of ewes to elevated ambient temperatures during pregnancy impairs fetal growth, severely reducing the weights of near-term fetuses (at 132 to 141 d of gestation, gD, term = gD 145–150) and new born lambs (Fig. 3). In the majority of studies, pregnant ewes were housed in climate-controlled chambers, and a circadian rhythm of temperature was enforced with a hotter day (ranges: 6 to18 h, 35 to 44 °C and temperature humidity index (THI) of 83.2 to 97.0) and cooler night (ranges: 6 to 18 h, 16 to 35 °C, THI of 38.3 to 83.5). When the data from these studies are combined (Fig. 3), they indicate that an increase in the duration of heat stress during pregnancy is associated with a greater reduction in fetal growth, with the possible exception of heat stress which persists throughout the entire pregnancy. This conclusion is supported by the study of Galan et al. [120] in which the effects of differing durations of maternal heat stress were directly compared. Relatively severe heat stress (18 h at 40 °C: 6 h at 35 °C) starting at gD35 reduced fetal weight in late gestation (~gD 130) by 45% in ewes exposed to heat for 55 d, and 74% in ewes exposed to heat for 80 d. Given the reduced fertility of ewes exposed to heat around mating, it is possible that the smaller reduction in lamb birthweight in ewes exposed to heat throughout pregnancy reflects a “survivor effect”, so only the best-adapted ewes conceive. The most severe effects occur when heat stress is imposed from mid-gestation. Reduced abdominal circumference is evident from gD 70, reductions in long bone lengths and skull width emerge at gD 80 and gD 90, respectively, and by late gestation, fetal sizes are 2 standard deviations or more below those of fetuses from thermoneutral ewes [121]. Consistent with these findings, newborn lambs and late gestation fetuses from heat-stressed ewes have altered morphology, including brain sparing [122–126], indicative of intrauterine growth-restriction.

Importantly, the impact of hyperthermia during pregnancy on fetal growth appears to be unaffected by litter size, with similar proportionate reductions in birthweight of singleton (↓1.16 kg) and twin lambs (↓0.99 kg) when heat stress is applied during the last two thirds of pregnancy [127]. The impacts of heat stress during pregnancy on birthweight are most severe when heat remains elevated throughout the day with no diurnal relief. Specifically, exposure to 32 °C for the last two thirds of pregnancy reduced birthweight by 1.76 kg and 0.75 kg when applied for 24 and 12 h per day, respectively [127].

### Lamb survival

**Fig. 4:**
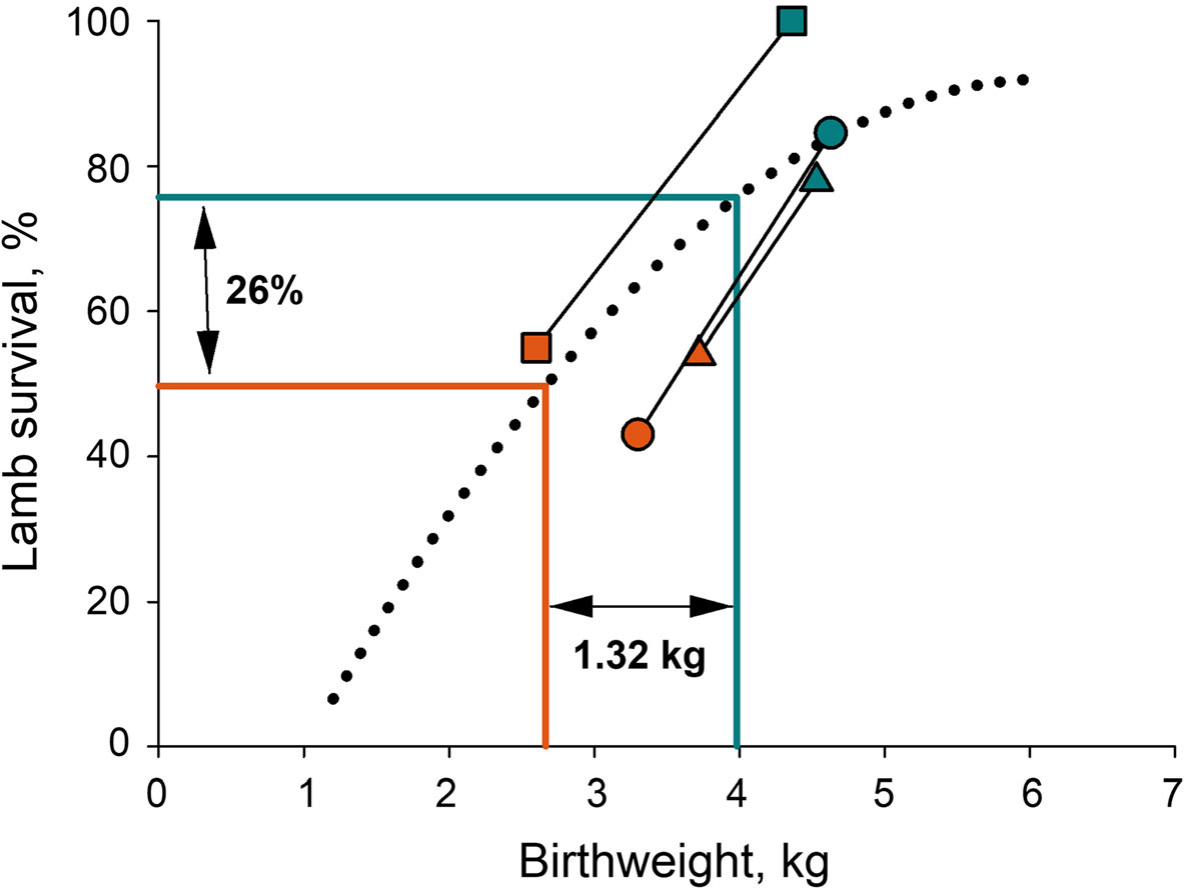
Predicted and reported impacts of maternal hyperthermia on lamb survival. Dotted line shows survival as a function of birthweight from Geenty et al. [139]. Drop lines show weighted mean birthweights derived from seven published studies in sheep and predicted survival for lambs from normothermic (blue line) and hyperthermic (orange line) pregnancies. Heat stress was imposed during the middle third of pregnancy [141], the final two-thirds of pregnancy [127, 134], the last third of pregnancy [128, 133, 135] or the entirety of pregnancy [26, 136]. Paired symbols show actual birthweight and survival data from normothermic (21–27 °C, blue symbols) and hyperthermic pregnancies (continuous at 32 °C, orange symbols) in comparably-fed ewes [127]

Birthweight is one of the main risk factors contributing to neonatal lamb deaths [137–139], with low birthweight lambs more susceptible to starvation-mismothering, hypothermia and death [140]. Only two studies have reported the impact of pregnancy hyperthermia on lamb viability and postnatal survival. Maternal hyperthermia throughout the last two thirds of pregnancy reduced survival by > 25% [127]. However, the impacts of elevated temperature (32 °C) during the last two thirds of pregnancy were more severe when hyperthermic conditions were maintained for 24 rather than 12 h per day (100% survival in thermoneutral conditions cf. 80% in 12 h/d heat stress and 55% in 24 h/d heat stress [127]). At least some of the impacts of maternal hyperthermia on lamb mortality is due to peripartum death with the proportion of lambs born alive falling from 97% in the field compared to 67% in ewes housed under 16 h 38 °C: 8 h 28–32 °C conditions [128]. We have used a model of the relationship between birthweight and lamb survival, based on records from 24,699 lambs across eight sites in southern Australia [139], to predict the impact of maternal hyperthermia during pregnancy. Maternal hyperthermia is predicted to reduce survival by 12.5% (heat-stress throughout pregnancy), 19% (heat-stressed only in middle third of pregnancy), 23% (heat-stressed only in last third of pregnancy), and 28.5% (heat-stressed throughout last two thirds of pregnancy), with a mean reduction in lamb survival of 26% after heat stress for at least a third of pregnancy (Fig. 4).

### Mechanisms for impacts of heat stress during pregnancy on fetal growth

#### Placental growth and function

Maternal hyperthermia impairs placental development and function with severity dependent on the stage/s of pregnancy during which ewes are exposed. During early to mid-pregnancy, there appears to be compensatory placental adaptations to maintain fetal growth. For example, when hyperthermia was induced from gD 35, the ratio of fetal to placental weight 15 to 20 d later was approximately 40% higher compared to control ewes even though fetal weight was not different from that of control ewes [122, 124]. Increased expression of insulin-like growth factors, placental growth factor and vascular endothelial growth factor (VEGF) in the placenta at gD 55 suggest mechanisms for initial compensatory mechanisms [123, 124]. Conversely, decreased VEGF receptor (VEGFR1) expression might impair angiogenic responses to VEGF [123]. However, ongoing heat stress after gD 55 reduces placental weights by approximately 33% to 36% (albeit non-significantly), and this is likely to underlie the 24% to 27% reduction in fetal weight which also occurs [122, 124]. Along with placental growth, maternal placental lactogen levels are reduced from gD 62 and circulating progesterone levels, primarily of placental origin, are reduced from gD 72 [122]. Placental effects of hyperthermia commencing later in pregnancy are less well characterised. Maternal hyperthermia throughout the last third of pregnancy reduces fetal weight by 30%, despite unchanged placental weight (Fig. 3), implying that this exposure substantially impairs placental function. Importantly, although the impacts are less severe than those of continuing heat stress, effects of heat stress in early to mid-pregnancy persist even when ewes return to thermoneutral conditions in mid-pregnancy. In ewes exposed to heat stress from gD 30 to gD 80, fetal and placental weights at gD 80 were reduced by 10% and 29%, respectively [129]. Although ewes were then returned to thermoneutral conditions, fetal and placental weights at gD 140 were reduced by 26% and 42%, respectively, compared to control ewes [129]. It is evident, that the negative impacts of maternal heat stress on fetal and placental growth and development are not recovered when ambient temperatures are lowered. Consequently, hyperthermic conditions occurring during the middle third of pregnancy, which is common when ewes are mated in spring or early summer, are likely to reduce lamb birthweights.

Reduced placental weights in late pregnancy reflect smaller rather than fewer cotyledons [130], with evidence of reduced placental cell division [131]. Decreased maternal circulating concentrations of placental lactogen from mid-gestation further suggests impaired development of binucleated trophoblast cells. Both active and passive transport across the placenta are markedly impaired at mid- to late-pregnancy following heat stress imposed from early pregnancy. In late gestation (gD 135), placental diffusion capacity was reduced by 47% in ewes exposed to hyperthermia from gD 45 until gD 120 [130], and by 47% to 52% after more severe hyperthermia [126, 142]. The reduction in placental diffusion capacity is proportional to fetal and placental weights [126, 142]. Oxygen extraction from maternal blood by the placenta was also lower after chronic hyperthermia, and although uterine oxygen delivery is proportional to fetal weight, lower umbilical blood flows result in fetal hypoxia, with 21% to 42% reductions in fetal arterial O_2_ saturation during late gestation [126, 130, 142]. Reduced umbilical blood flow is associated with elevated markers of resistance to blood flow, apparent by gD 80 to 90 [143], implicating resistance to blood flow within the placental bed. Placental transport of glucose is likewise impaired in heat-stressed ewes, reducing fetal plasma glucose by 37% [126]. Placental uptake and transfer of the branched-chain amino acid analogue ACP, measuring active system _L_-amino acid transport, is also impaired in ewes after chronic heat stress [125].

#### Maternal nutrition

**Fig. 5:**
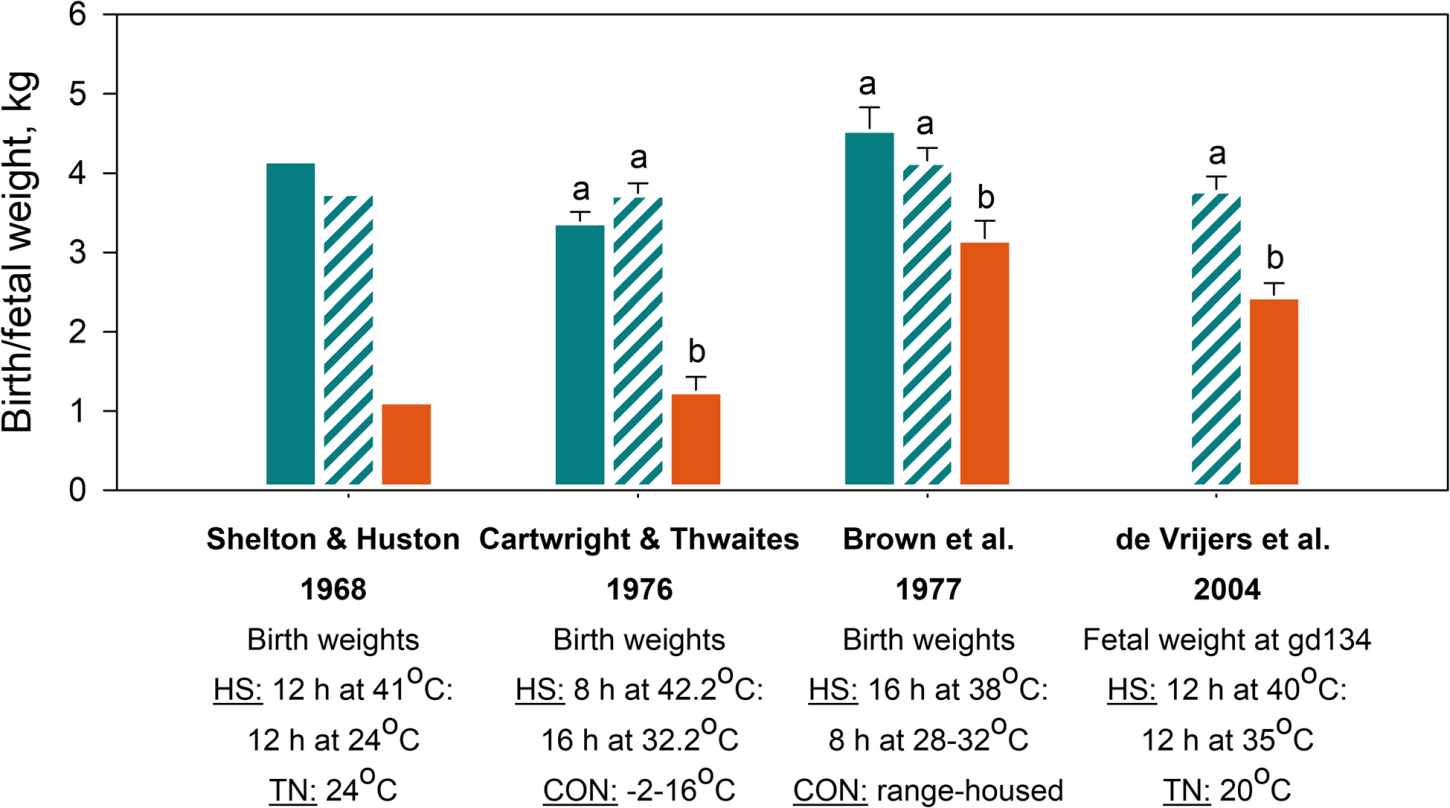
Reduced feed intake is not the major mechanism causing reduced birthweight in heat-stressed ewes. Ewes were housed in non-heat-stressed conditions (blue bars) and either fed *ad libitum* (solid orange bars) or pair-fed (striped bars) to ewes housed in hyperthermic conditions. Data are means, error bars indicate SEM, and significant differences are indicated by differing superscripts where stated in the source. Data derived from [127, 128, 134]. HS, Heat stress; TN, thermoneutral; Con, Control

Nutrition during pregnancy is a key determinant of placental and fetal growth [144, 145], and undernutrition during the last two thirds of pregnancy can impair both [146]. Heat stress reduces voluntary feed intake of pregnant ewes by 6% to 34% [127, 132, 133]. However, most studies suggest the negative effects of heat stress on placental and fetal growth are largely independent of nutrient intake [127, 128, 132, 133]. Specifically, when the feed intake of ewes housed in thermoneutral conditions was matched with that of heat-stressed ewes, the effects of heat stress on birthweight were far greater than those resulting from reduced feed intake alone. Compared with *ad libitum-*fed ewes housed under thermoneutral conditions, birthweight was substantially reduced (average ↓1850 g) in heat-stressed ewes but was not significantly reduced in thermoneutral ewes which were pair-fed to the heat-stressed group (average ↓156 g; Fig. 5). Similarly, fetal weights were reduced in heat-stressed ewes (24% to 27% reduction at gD 90 to 93 and 23% to 53% reduction at gD134), compared with their pair-fed, thermoneutral counterparts [122, 124, 125]. It is therefore clear that reduced maternal feed intake is not the primary cause of heat stress-induced reductions in birthweight.

#### Temperature load

Chronic hyperthermia increases ewe core temperature by 0.6 to 2.0 °C [122–124, 126, 128, 132, 134, 141, 147, 148]. The metabolically active fetus produces heat, 85% of which is lost via the circulatory systems of the umbilical cord and placenta [149]. The remaining 15% dissipates across the amniotic fluid and into the uterine wall [149], a process which is facilitated by fetal temperatures exceeding those of the dam. Under thermoneutral conditions, the difference between fetal and maternal temperature (feto-maternal temperature gradient) is approximately 0.4 to 0.7 °C depending on the stage of pregnancy [150–152]. Ewe core temperature and fetal temperature both rise in response to short periods (1 to 4 h) of heat exposure; however, at progressively higher ambient temperatures the feto-maternal gradient decreases. Four hours at 35 °C decreased the feto-maternal temperature gradient but still allowed maintenance of a positive gradient from fetus to dam [152]. However, after 2.5 h at 40 °C, fetal and maternal temperatures equalised and 1 h of 42 °C produced a negative feto-maternal gradient, likely due to changes in uterine and umbilical blood flows [150–153]. Fetal capacity to dissipate heat is positively related to the rate of blood flow away from the placenta and uterus as well as umbilical blood flow [150–152]. Under moderate heat stress that increases maternal temperature by < 2 °C, and when hyperthermia is induced gradually, uterine blood flow increases [153] effectively increasing fetal heat loss via the circulation. In contrast, 1 h of severe heat stress (40 to 42 °C) significantly reduced uterine and umbilical blood flow, with the former persisting for over an hour [147, 150]. It seems likely that accumulation of heat in the fetus under severe or rapid heat stress suppresses fetal metabolic rate to reduce heat generation, and that this may contribute to impaired fetal growth, particularly under conditions of extreme or rapid increases in heat.

Interestingly, changes in core temperature in response to elevated temperature vary between ewes [154, 155] presumably reflecting differences in their tolerance, and thermoregulatory response, to temperature elevations. There are significant negative relationships between ewe rectal temperature during periods of high temperature and both lamb birthweight [133, 153, 154] and placental weight [155]. Depending on the year, lamb birthweight decreased by 1.42 to 1.59 kg/°C increase in ewe rectal temperature in response to high ambient temperature, and ewes which reared a lamb had lower rectal temperatures than those which failed to do so [154].

#### Progeny performance and thermotolerance

The negative impact of exposure to heat stress in utero on lamb birthweight and, potentially, postnatal survival is clear. However, to the best of our knowledge, the impact of in utero heat stress on postnatal physiology, performance and thermotolerance has not been investigated in sheep. In dairy cattle, heat stress during the peri-conception period or pregnancy alters the physiology and performance of offspring [156–158]. Negative impacts include impaired immunoglobulin absorption and passive immunity in calves [159, 160], as well as reduced milk production and fertility development later in life [157, 160, 161]. Interestingly, there is also preliminary evidence of lower core temperature [161, 162] and improved thermo-tolerance [162] in adult cows which experienced in utero heat stress for the last 50 d prior to birth [162]. In contrast, in utero heat stress impairs rather than improves progeny thermotolerance in pigs, increasing the postnatal core temperature set point, and reducing their ability to thermoregulate [163, 164]. It is also evident from a number of studies in pigs that in utero heat stress can impair reproductive development of male and female progeny, alter body composition, and impair milk yield (see review by Johnson & Baumgard [165]). It is concluded that in utero heat stress has the potential to alter progeny phenotype sufficiently to alter their productivity and ability to cope with extreme thermal environment.

#### Mammogenesis

The impact of heat stress during pregnancy on mammary function and, thus, colostrum and milk production and pre-weaning lamb growth has not been investigated in sheep. In dairy cattle, heat stress during the last 50 d of pregnancy compromises mammary development and decreases colostrum and milk production (average decline of 3.6 kg/d or 10.3%; Dado-Senn et al. [166], Ouellet et al. [158]), likely due to altered placental hormone production and reduced dry matter intake [167]. Placental lactogen promotes mammary development and milk production in sheep [168] and the decreased placental lactogen evident by mid-pregnancy in heat-stressed ewes [122] is therefore likely to impair mammary development. In the absence of heat stress, a 40% reduction in feed intake during the last two thirds of pregnancy decreased mammary weight and colostrum production by 20% and 43%, respectively [169]. A 50% reduction in feed intake during the last 42 d of pregnancy reduced the volume of colostrum and milk produced by 68% and 33%, respectively [170]. It is, therefore, possible that heat stress-induced reductions in voluntary feed intake may also impair mammary development and colostrum production.

## Heat stress during lactation

The sensitivity of ewes to thermal stress is higher during lactation than during late pregnancy, reflecting the high metabolic, heat-producing load of milk production [20]. However, data describing the impact of elevated temperature and humidity on lactation are sparse for wool- and meat-producing sheep breeds, although thermal stress during lactation decreases milk production of dairy cattle [156] and dairy sheep [171]. Dairy cows exposed to mild heat stress during lactation produce 25 to 40% less milk, primarily due to heat induced reductions in feed intake, alterations in metabolism and impaired mammary gland development [156]. In dairy sheep, Ramón et al. [172] reported that optimal daily temperatures for milk production are 10 to 22 °C, and that modest variations in temperature outside this range were sufficient to reduce milk yield. Milk volume is reduced by 15% when maximum temperatures exceeded 21 to 24 °C, and by 20% at THI of 72 to 75 compared with 60 to 65 (see review by Sevi and Caroprese [171]. Lactating dairy goats are also affected by thermal stress, eating 29% less feed, drinking 41% more water and producing 8% less milk when exposed to thermal stress for 32 d [173]. Given this evidence, rising temperatures during spring may impair lactation performance of ewes under field conditions, impairing pre-weaning growth of their lambs.

## Interaction of heat stress with other environmental stressors

Most studies examining the effects of heat stress on ewe reproductive performance have used housed animals in environmental chambers where the effects of heat stress can be examined with minimal involvement of confounding factors. However, the potential for other stressors to influence sheep reproduction is substantial. Heat stress reduces feed intake [33, 69, 174], and ewes which are exposed to heat stress in the field are not only exposed to nutritional deprivation and loss of appetite, they also experience the stress of having to walk long distances to access both food and water [175]. During the hotter periods of the year, available food is typically dry and highly fibrous, dietary characteristics known to increase internal heat load [2]. These secondary stressors can produce responses that potentially confound and/or exacerbate the effects of heat stress per se on reproductive performance, and are further exacerbated by behaviours sheep adopt to reduce heat load and aid in temperature dissipation. Sheep cope with heat stress conditions by changing their use of the landscape including spending more time in shaded areas and at water points (reviewed by Al-Dawood [5]). Thermal conditions which influence when and where animals are active can be costly in regard to energy intake, especially if heat load constrains foraging time or if foraging is shifted to areas that provide poorer feed quality and/or availability.

Despite their importance, there is very little information on how these stressors interact to affect reproduction in sheep. Spies et al. [176] examined the effects of forced exercise (30 or 60 min daily) and ambient heat stress applied immediately before and after oestrus. Exercise stress and heat stress independently reduced fertilisation rates with the worst result being obtained when the two stressors were combined. Other studies [177, 178] have found that combined stressors (heat and nutrition) adversely affect reproductive performance but in neither study could the individual effects be separated. Sejian et al. [175] subsequently examined the effects of ambient heat stress combined with both a restricted diet (30% intake) and walking 14 km per day on the reproductive performance of Malpura sheep in India. All parameters examined were adversely affected by this combined stress, including a reduction in the number of ewes which came into oestrus from 66.7% to 41.7%, shortening of oestrus duration from 32 h to 14.4 h, lowering of conception rates from 83.3% to 50.0% (*P* > 0.05), and significantly altered steroid levels. It is, therefore, plausible that even if naturally occurring periods of high ambient temperature are not as high or as sustained as those applied in studies using environmental chambers, the additive effects of exercise and reduced feed quality together with heat may impair reproduction.

## Conclusions

**Fig. 6:**
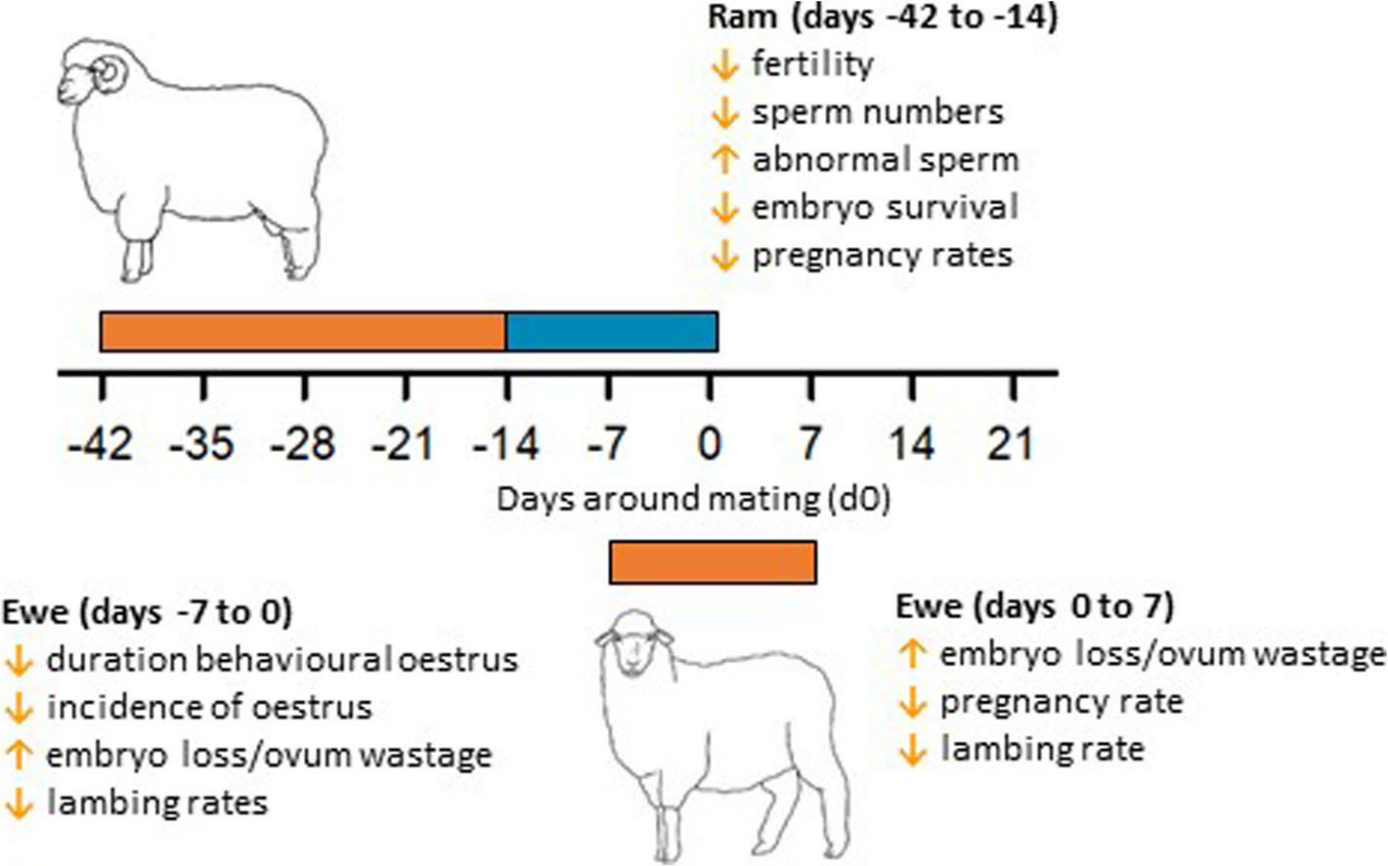
Summarised effects of heat stress on ram and ewe fertility prior to and during the mating period. Timing of normothermic (blue) and hyperthermic (orange) temperatures prior to mating

**Fig. 7:**
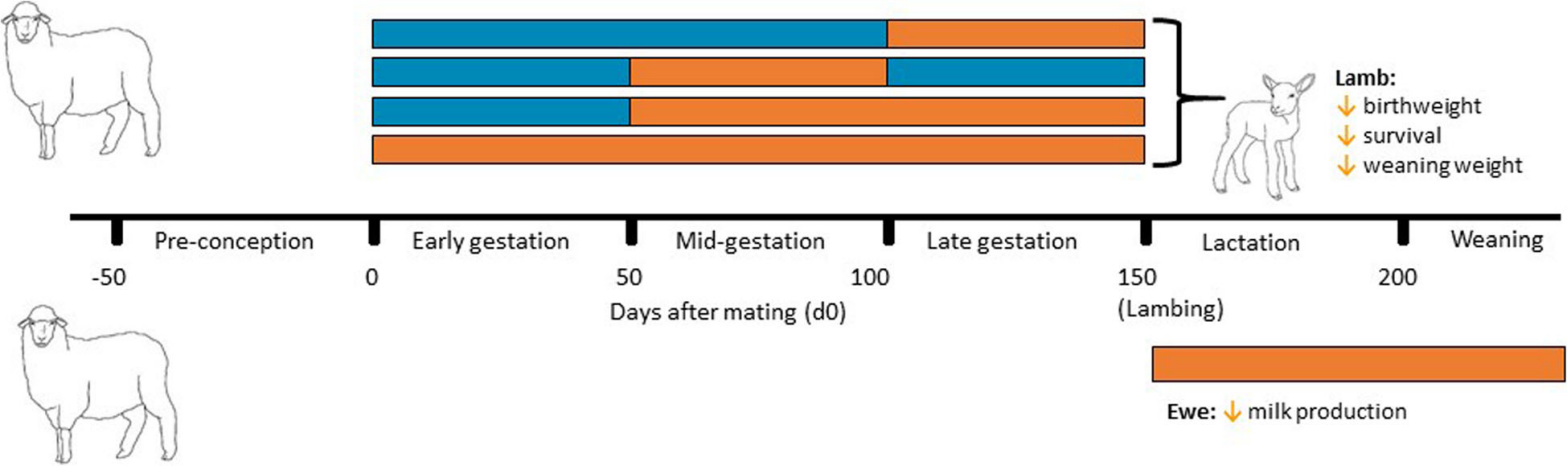
Summarised effects of heat stress during gestation and lactation on lamb growth, development and survival, as well as milk production. Timing of normothermic (blue) and hyperthermic (orange) temperatures during gestation and lactation

It is clear that short- and long-term exposure to heat stress significantly impairs the fertility and fecundity of ewes, and decreases semen production and quality of rams, resulting in fewer lambs born per ewe mated (Fig. 6 and Fig. 7). Furthermore, reductions in lamb birthweight and ewe mammary development resulting from heat stress during pregnancy, are likely to significantly decrease lamb survival and weight at weaning (Fig. 6 and Fig. 7).

The impact of heat stress on ewe fertility is most severe when experienced from 5 d before oestrus until 5 d after oestrus. Heat stress imposed for 5 d before oestrus impairs fertilisation rates by nearly 60%, with a smaller drop (~ 30%) seen when heat stress commences at oestrus. Heat stress commencing a day or more after oestrus does not impair fertilisation, but can reduce lambing rates by up to 80% due to embryo loss. Embryo losses are greater the sooner heat stress commences after oestrus, with most embryos lost prior to d 12 post-mating. It is likely that these losses result from compromised oocyte maturation in association with alterations in follicle growth and function and in the production of ROS species. Equally, the impact of heat stress on the oviduct environment is a likely cause of fertilisation failure and early embryo death but this has not been thoroughly examined. Importantly, the relevant temperature controlled studies which have revealed the nature of the relationship between heat stress and ewe fertility were conducted at ≥32 °C, thus supporting evidence from field studies that ewe fertility is negatively correlated with the number of days ≥32 °C which occur during the mating period.

Studies using environmental chambers also demonstrate that heat stress impairs spermatogenesis and semen quality, and that fertility is reduced when ewes are mated with heat-stressed rams. However, the contribution of the ram to impaired mating outcomes during periods of heat stress in the field remains poorly quantified given the delayed impact of heat stress on sperm quality, the variation in the sensitivity of individual rams, and the potential ability of less-affected rams to mask the performance of poorer performing individuals in flock-mating situations.

It is clear that sustained exposure to high temperatures (32 to 41 °C) during pregnancy reduces lamb birthweight and viability, thus increasing neonatal mortality. Heat-induced fetal growth restriction occurs independently of feed intake. Instead, short and long-term physiological changes designed to maintain core temperature under heat stress conditions impair placental development and fetal growth. Of particular concern is the impact that relatively modest heat stress (32 °C) has on lamb birthweight, with a significant proportion of the global sheep population experiencing prolonged periods of 30 to 35 °C during pregnancy. Furthermore, these negative impacts of maternal heat stress on conceptus development are not recovered when ambient temperatures are lowered later in pregnancy. Although data are currently lacking, the potential for heat stress during pregnancy to impact lamb survival under field conditions is high and may already contribute to high rates of lamb mortality globally.

The use of studies involving environmental chambers to identify the critical period(s) and mechanisms whereby heat stress affects ewe and ram reproduction provides a useful basis for the development of strategies for the management of sheep flocks exposed to high ambient temperatures. Although the majority of these intensive studies were conducted over two decades ago, evidence that heat stress continues to impair ewe fertility is provided from three recent studies [10–12]. Despite this, there is minimal data describing the impacts of the climate experienced by pasture and rangeland sheep on their homeothermy, behaviour, resource use and reproduction. This applies to ewes at all stages of the breeding cycle (before joining through to weaning), rams, as well as the progeny born to heat-stressed ewes and rams. Currently, the ability to ameliorate the impact of heat stress on sheep through changes in husbandry practices, the environment and genetic selection is limited. However, as global warming intensifies and the likelihood that multiple periods of the reproductive cycle will be affected by prolonged heat stress events increases, there is growing urgency to develop intervention strategies to ameliorate the reproductive impacts of heat stress.

## Data Availability

Not applicable.

## Abbreviations

d: Day
g: Gram
gD: Day of gestation
h: Hour
kg: Kilogram
LH: Luteinising hormone
THI: Temperature humidity index

## References

[1] Collier RJ, Renquist BJ, Xiao Y. A 100-year review: stress physiology including heat stress. J Dairy Sci. 2017;100 10.3168/jds.2017-13676. 10.3168/jds.2017-13676 29153170

[2] Silanikove N. Effects of heat stress on the welfare of extensively managed domestic ruminants. Livestock Prod Sci. 2000;67 https://doi.org/10.1016/S0301-6226(00)00162-7.

[3] Bernabucci U, Lacetera N, Baumgard LH, Rhoads RP, Ronchi B, Nardone A. Metabolic and hormonal acclimation to heat stress in domesticated ruminants. Animal. 2010;4 10.1017/S175173111000090X. 10.1017/S175173111000090X 22444615

[4] Marai IFM, El-Darawany AA, Fadiel A, Abdel-Hafez MAM. Physiological traits as affected by heat stress in sheep: a review. Small Ruminant Res. 2007;71 10.1016/j.smallrumres.2006.10.003.

[5] Al-Dawood A. Towards heat stress management in small ruminants: a review. Ann Animal Sci. 2017;17 10.1515/aoas-2016-0068.

[6] Crimp SJ, Stokes CJ, Howden SM, Moore AD, Jacobs B, Brown PR, et al. Managing Murray-Darling Basin livestock systems in a variable and changing climate: challenges and opportunities. Rangeland J. 2010;32 10.1071/RJ10039.

[7] Henry B, Charmley E, Eckard R, Gaughan JB, Hegarty R. Livestock production in a changing climate: adaptation and mitigation research in Australia. Crop Pasture Sci. 2012;63 10.1071/CP11169.

[8] Eldridge DJ, Beecham G. The impact of climate variability on land use and livelihoods in Australia’s rangelands. In: Gaur MK, R SV, editors. Climate Variability Impacts on Land Use and Livelihoods in Drylands. First ed: Springer, Cham; 2018. p. 293–315.

[9] Lindsay DR, Knight TW, Smith JF, Oldham CM. Studies in ovine fertility in agricultural regions of Western Australia: ovulation rate, fertility and lambing performance. Australian J Agricultural Res. 1975;26 10.1071/AR9750189.

[10] Kleemann DO, Walker SK. Fertility in South Australian commercial Merino flocks: relationships between reproductive traits and environmental cues. Theriogenol. 2005;63 10.1016/j.theriogenology.2004.09.052. 10.1016/j.theriogenology.2004.09.052 15910923

[11] Palacios C, Abecia JA. Meteorological variables affect fertility rate after intrauterine artificial insemination in sheep in a seasonal-dependent manner: a 7-year study. Int J Biometeorol. 2015;59 10.1007/s00484-014-0872-y. 10.1007/s00484-014-0872-y 25056126

[12] Santolaria P, Yániz J, Fantova E, Vicente-Fiel S, Palacín I. Climate factors affecting fertility after cervical insemination during the first months of the breeding season in Rasa Aragonesa ewes. Int J Biometeorol. 2014;58 10.1007/s00484-013-0770-8. 10.1007/s00484-013-0770-8 24317703

[13] AusCover. Australian Gridded Climate Data Australia: AusCover facility of the Terrestrial Ecosystem Research Network; 2019 [Available from: http://www.auscover.org.au/datasets/australian-gridded-climate-data/.

[14] van Wettere WHEJ, Culley S, Gatford KG, Kind KL, Lee S, Leu ST, et al. Effects of heat stress, and predicted climate change scenarios on reproductive performance of the Australian sheep flock. North Sydney, NSW: The University of Adelaide and The South Australian Research and Development Institute; 2019. Contract No.: L.LSM.0024.

[15] Dutt RH. Detrimental effects of high ambient temperature on fertility and early embryo survival in sheep. Int J Biometeorol. 1964;8 10.1007/BF02186927. 10.1007/BF02186927 5888798

[16] Dutt RH. Critical period for early embryo mortality in ewes exposed to high ambient temperature. J Anim Sci. 1963;22 10.2527/jas1963.223713x.

[17] Dutt RH, Ellington EF, Carlton WW. Fertilization rate and early embryo survival in sheared and unsheared ewes following exposure to elevated air temperature. J Anim Sci. 1959;18 10.2527/jas1959.1841308x.

[18] Thwaites CJ. Short term heat stress and embryo mortality in the ewe. Aust J Exp Agr. 1971;11 10.1071/EA9710265.

[19] Romo-Barron CB, Diaz D, Portillo-Loera JJ, Romo-Rubio JA, Jimenez-Trejo F, Montero-Pardo A. Impact of heat stress on the reproductive performance and physiology of ewes: a systematic review and meta-analyses. Int J Biometeorol. 2019;63 10.1007/s00484-019-01707-z. 10.1007/s00484-019-01707-z 30888508

[20] Abdalla EB, Kotby EA, Johnson HD. Physiological responses to heat-induced hyperthermia of pregnant and lactating ewes. Small Ruminant Res. 1993;11 10.1016/0921-4488(93)90145-8.

[21] Rich TD, Alliston CW. Influence of programmed circadian temperature changes on the reproductive performance of ewes. J Anim Sci. 1970;30 10.2527/jas1970.306966x. 10.2527/jas1970.306966x 5422009

[22] Naqvi SMK, Maurya VP, Gulyani R, Joshi A, Mittal JP. The effect of thermal stress on superovulatory response and embryo production in Bharat Merino ewes. Small Ruminant Res. 2004;55 10.1016/j.smallrumres.2004.02.009.

[23] Sawyer GJ, Lindsay DR, Martin GB. The influence of radiant heat load on reproduction in the Merino ewe: 3. Duration of oestrus, cyclical oestrous activity, plasma progesterone, LH levels and fertility of ewes exposed to high temperatures before mating. Aust J Agricultural Res. 1979;30 10.1071/AR9791151.

[24] Indu S, Sejian V, Naqvi SMK. Impact of simulated semiarid tropical environmental conditions on growth, physiological adaptability, blood metabolites and endocrine responses in Malpura ewes. Anim Prod Sci. 2015;55 10.1071/AN14085.

[25] Sawyer GJ. The influence of radiant heat load on reproduction in the Merino ewe: 2. The relative effects of heating before and after insemination. Aust J Agricultural Res. 1979a;30 10.1071/AR9791143.

[26] Yeates NTM. The effect of high air temperature on pregnancy and birth weight in Merino sheep. Aust J Agricultural Res. 1956;7 10.1071/AR9560435.

[27] Smith ID. Reproduction in Merino sheep in tropical Australia. Aust Vet J. 1964;40 10.1111/j.1751-0813.1964.tb01723.x.

[28] Smith ID. Reproductive wastage in a Merino flock in central western Queensland. Aust Vet J. 1962;38 10.1111/j.1751-0813.1962.tb04000.x.

[29] Lamond DR, Wells KE, Miller SJ. Study of a breeding problem in Merino ewes in central Queensland. Aust Vet J. 1963;39 10.1111/j.1751-0813.1963.tb04333.x.

[30] Fowler DG (1984). Reproductive behaviour of rams. In: Lindsay D, Pearce D, editors. Reproduction in sheep. First ed.

[31] Schillo KK, Alliston CW, Malven PV. Plasma concentrations of luteinizing hormone and prolactin in the ovariectomized ewe during induced hyperthermia. Biol Reprod. 1978;19 10.1095/biolreprod19.2.306. 10.1095/biolreprod19.2.306 719089

[32] Wise ME, Armstrong DV, Huber JT, Hunter R, Wiersma F. Hormonal alterations in the lactating dairy cow in response to thermal stress. J Dairy Sci. 1988;71 10.3168/jds.S0022-0302(88)79834-3. 10.3168/jds.S0022-0302(88)79834-3 3183143

[33] Ozawa M, Tabayashi D, Latief TA, Shimizu T, Oshima I, Kanai Y. Alterations in follicular dynamics and steroidogenic abilities induced by heat stress during follicular recruitment in goats. Reproduction. 2005;129 10.1530/rep.1.00456. 10.1530/rep.1.00456 15855625

[34] Badinga L, Thatcher WW, Diaz T, Drost M, Wolfenson D. Effect of environmental heat stress on follicular development and steroidogenesis in lactating Holstein cows. Theriogenology. 1993;39 10.1016/0093-691X(93)90419-6. 10.1016/0093-691x(93)90419-6 16727254

[35] Wilson SJ, Kirby CJ, Koenigsfeld AT, Keisler DH, Lucy MC. Effects of controlled heat stress on ovarian function of dairy cattle. 2. Heifers. J Dairy Sci. 1998;81 10.3168/jds.S0022-0302(98)75789-3. 10.3168/jds.S0022-0302(98)75789-3 9749377

[36] Wilson SJ, Marion RS, Spain JN, Spiers DE, Keisler DH, Lucy MC. Effects of controlled heat stress on ovarian function of dairy cattle. 1. Lactating cow. J Dairy Sci. 1998;81 10.3168/jds.S0022-0302(98)75788-1. 10.3168/jds.S0022-0302(98)75788-1 9749376

[37] Wolfenson D, Lew BJ, Thatcher WW, Graber Y, Meidan R. Seasonal and acute heat stress effects on steroid production by dominant follicles in cows. Animal Reprod Sci. 1997;47 10.1016/S0378-4320(96)01638-7. 10.1016/s0378-4320(96)01638-7 9233502

[38] Wolfenson D, Thatcher WW, Badinga L, Savi JD, Meidan R, Lew BJ, et al. Effect of heat stress on follicular development during the estrous cycle in lactating dairy cattle. Biol Reprod. 1995;52 10.1095/biolreprod52.5.1106. 10.1095/biolreprod52.5.1106 7626710

[39] Hill TG, Alliston CW. Effects of thermal stress on plasma concentrations of luteinizing hormone, progesterone, prolactin and testosterone in the cycling ewe. Theriogenology. 1981;15 10.1016/S0093-691X(81)80008-8. 10.1016/s0093-691x(81)80008-8 16725578

[40] Sheikheldin MA, Howland BE, Palmer WM. Effects of heat stress on serum progesterone in cyclic ewes and on progesterone and cortisol response to ACTH in ovariectomized ewes. J Reprod Fertility. 1988;84 10.1530/jrf.0.0840521. 10.1530/jrf.0.0840521 2848944

[41] Alliston CW, Ulberg LC. Early pregnancy loss in sheep at ambient temperatures of 70 and 90°F as determined by embryo transfer. J Anim Sci. 1961;20 10.2527/jas1961.203608x.

[42] Trout JP, McDowell LR, Hansen PJ. Characteristics of the estrous cycle and antioxidant status of lactating Holstein cows exposed to heat stress. J Dairy Sci. 1998;81 10.3168/jds.S0022-0302(98)75685-1. 10.3168/jds.S0022-0302(98)75685-1 9621225

[43] Roth Z, Meidan R, Braw-Tal R, Wolfenson D. Immediate and delayed effects of heat stress on follicular development and its association with plasma FSH and inhibin concentration in cows. J Reprod Fertility. 2000;120 10.1530/jrf.0.1200083. 11006149

[44] Wolfenson D, Roth Z, Meidan R. Impaired reproduction in heat-stressed cattle: basic and applied aspects. Animal Reprod Sci. 2000;60–61 10.1016/s0378-4320(00)00102-0. 10.1016/s0378-4320(00)00102-0 10844222

[45] Hansen PJ. Effects of heat stress on mammalian reproduction. Philosophical Transactions of the Royal Society of London Series B: Biol Sci. 2009;364 10.1098/rstb.2009.0131. 10.1098/rstb.2009.0131 PMC2781849 19833646

[46] Wiebold JL. Embryonic mortality and the uterine environment in first-service lactating dairy cows. J Reprod Fertility. 1988;84 10.1530/jrf.0.0840393. 10.1530/jrf.0.0840393 3199356

[47] Ryan DP, Prichard JF, Kopel E, Godke RA. Comparing early embryo mortality in dairy cows during hot and cool seasons of the year. Theriogenology. 1993;39 10.1016/0093-691X(93)90257-6. 10.1016/0093-691x(93)90257-6 16727249

[48] Sartori R, Sartor-Bergfelt R, Mertens SA, Guenther JN, Parrish JJ, Wiltbank MC. Fertilization and early embryonic development in heifers and lactating cows in summer and lactating and dry cows in winter. J Dairy Sci. 2002;85 10.3168/jds.S0022-0302(02)74367-1. 10.3168/jds.S0022-0302(02)74367-1 12487447

[49] Roth Z, Meidan R, Shaham-Albalancy A, Braw-Tal R, Wolfenson D. Delayed effect of heat stress on steroid production in medium-sized and preovulatory bovine follicles. Reproduction. 2001;121 10.1530/rep.0.1210745. 11427162

[50] Paes VM, Vieira LA, Correia HHV, Sa NAR, Moura AAA, Sales AD, et al. Effect of heat stress on the survival and development of *in vitro* cultured bovine preantral follicles and on *in vitro* maturation of cumulus-oocyte complex. Theriogenology. 2016;86 10.1016/j.theriogenology.2016.03.027. 10.1016/j.theriogenology.2016.03.027 27125691

[51] Maya-Soriano MJ, Taberner E, López-Béjar M. Retinol improves *in vitro* oocyte nuclear maturation under heat stress in heifers. Zygote. 2013;21 10.1017/S0967199412000135. 10.1017/S0967199412000135 22785151

[52] Nabenishi H, Takagi S, Kamata H, Nishimoto T, Morita T, Ashizawa K, et al. The role of mitochondrial transition pores on bovine oocyte competence after heat stress, as determined by effects of cyclosporin A. Mole Reprod Development. 2012;79 10.1002/mrd.21401. 10.1002/mrd.21401 22128015

[53] Cebrian-Serrano A, Salvador I, Raga E, Dinnyes A, Silvestre MA. Beneficial effect of melatonin on blastocyst *in vitro* production from heat-stressed bovine oocytes. Reprod Domestic Animals. 2013;48 10.1111/rda.12154. 10.1111/rda.12154 23458773

[54] Ryle M. Early reproductive failure of ewes in a hot environment I. Ovulation rate and embryonic mortality. J Agricultural Sci. 1961;57 10.1017/S0021859600049947.

[55] Sawyer GJ. The influence of radiant heat load on reproduction in the Merino ewe: 1. The effect of timing and duration of heating. Aust J Agricultural Res. 1979b;30 10.1071/AR9791133.

[56] Alliston CW, Egli GE, Ulberg LC. Loss of potential young in the ewe due to high ambient temperature. J Appl Physiol. 1961;16 10.1152/jappl.1961.16.2.253. 10.1152/jappl.1961.16.2.253 13682606

[57] Lenz RW, Ball GD, Leibfried ML, Ax RL, First NL. *In vitro* maturation and fertilization of bovine oocytes are temperature-dependent processes. Biol Reprod. 1983;29 10.1095/biolreprod29.1.173. 10.1095/biolreprod29.1.173 6615963

[58] Payton RR, Romar R, Coy P, Saxton AM, Lawrence JL, Edwards JL. Susceptibility of bovine germinal vesicle-stage oocytes from antral follicles to direct effects of heat stress *in vitro*. Biol Reprod. 2004;71 10.1095/biolreprod.104.029892. 10.1095/biolreprod.104.029892 15201201

[59] Roth Z, Hansen PJ. Disruption of nuclear maturation and rearrangement of cytoskeletal elements in bovine oocytes exposed to heat shock during maturation. Reproduction. 2005;129 10.1530/rep.1.00394. 10.1530/rep.1.00394 15695618

[60] Wang J-Z, Sui H-S, Miao D-Q, Liu N, Zhou P, Ge L, et al. Effects of heat stress during *in vitro* maturation on cytoplasmic versus nuclear components of mouse oocytes. Reproduction. 2009;137 10.1530/REP-08-0339. 10.1530/REP-08-0339 19029342

[61] Ahmadi E, Nazari H, Hossini-Fahraji H. Low developmental competence and high tolerance to thermal stress of ovine oocytes in the warm compared with the cold season. Tropical Animal Health Prod. 2019;51 10.1007/s11250-019-01854-w. 10.1007/s11250-019-01854-w 30840214

[62] Gharibzadeh Z, Riasi A, Ostadhosseini S, Hosseini SM, Hajian M, Nasr-Esfahani MH. Effects of heat shock during the early stage of oocyte maturation on the meiotic progression, subsequent embryonic development and gene expression in ovine. Zygote. 2015;23 10.1017/S0967199414000203. 10.1017/S0967199414000203 24964001

[63] Matsuzuka T, Sakamoto N, Ozawa M, Ushitani A, Hirabayashi M, Kanai Y. Alleviation of maternal hyperthermia-induced early embryonic death by administration of melatonin to mice. J Pineal Res. 2005;39 10.1111/j.1600-079X.2005.00260.x. 10.1111/j.1600-079X.2005.00260.x 16150100

[64] Matsuzuka T, Ozawa M, Nakamura A, Ushitani A, Hirabayashi M, Kanai Y. Effects of heat stress on the redox status in the oviduct and early embryonic development in mice. J ReprodDev. 2005;51 10.1262/jrd.16089. 10.1262/jrd.16089 15699582

[65] Sakatani M, Yamanaka K, Kobayashi S, Takahashi M. Heat shock-derived reactive oxygen species induce embryonic mortality in *in vitro* early stage bovine embryos. J Reprod Dev. 2008;54 10.1262/jrd.20017. 10.1262/jrd.20017 18762719

[66] Barakat IAH, Khalil WKB, Al-Himaidi AR. Curcacycline A and B modulate apoptosis induced by heat stress in sheep oocytes during *in vitro* maturation. Small Ruminant Res. 2016;136 10.1016/j.smallrumres.2016.01.020.

[67] Ozawa M, Hirabayashi M, Kanai Y. Developmental competence and oxidative state of mouse zygotes heat-stressed maternally or *in vitro*. Reproduction. 2002;124 10.1530/rep.0.1240683. 10.1530/rep.0.1240683 12417007

[68] Thwaites CJ. Embryo mortality in the heat stressed ewe: 2. Application of hot-room results to field conditions. J Reprod Fertility. 1969;19 10.1530/jrf.0.0190255. 10.1530/jrf.0.0190255 5815726

[69] Thwaites CJ. Embryo mortality in the heat stressed ewe: 1. The influence of breed. J Reprod Fertility. 1967;14 10.1530/jrf.0.0140005. 10.1530/jrf.0.0140005 6033308

[70] Staigmiller RB, Moor RM. Effect of follicle cells on the maturation and developmental competence of ovine oocytes matured outside the follicle. Gamete Res. 1984;9 10.1002/mrd.1120090211.

[71] Woody CO, Ulberg LC. Viability of one-cell sheep ova as affected by high environmental temperature. J Reprod Fertility. 1964;7 10.1530/jrf.0.0070275. 10.1530/jrf.0.0070275 14180721

[72] Setchell BP. The Parkes Lecture: Heat and the testis. J Reprod Fertility. 1998;114 10.1530/jrf.0.1140179. 10.1530/jrf.0.1140179 10070346

[73] Waites GMH, Moule GR. Relation of vascular heat exchange to temperature regulation in testis of ram. J Reprod Fertility. 1961;2 10.1530/jrf.0.0020213. 10.1530/jrf.0.0020213 14004433

[74] Waites GMH, Setchell BP. Effect of local heating on blood flow and metabolism in testis of conscious ram. J Reprod Fertility. 1964;8 10.1530/jrf.0.0080339. 10.1530/jrf.0.0080339 14248594

[75] Fowler DG. Skin folds and Merino breeding. 5. Variations in scrotal, testis and rectal temperatures as affected by site of measurement, acclimatization to heat and degree of skin fold. Aust J Exp Agr. 1968;8 10.1071/EA9680125.

[76] Coulter GH, Senger PL, Bailey DR. Relationship of scrotal surface temperature measured by infrared thermography to subcutaneous and deep testicular temperature in the ram. J Reprod Fertility. 1988;84 10.1530/jrf.0.0840417. 10.1530/jrf.0.0840417 3199358

[77] Arman C, Quintana Casares PI, Sanchez-Partida LG, Setchell BP. Ram sperm motility after intermittent scrotal insulation evaluated by manual and computer-assisted methods. Asian J Androl. 2006;8 10.1111/j.1745-7262.2006.00145.x. 10.1111/j.1745-7262.2006.00145.x 16763716

[78] Murray PJ, Rowe JB, Pethick DW. Effect of season and nutrition on scrotal circumference of Merino rams. Aust J Exp Agr. 1991;31 10.1071/EA9910753.

[79] Marai IFM, El-Darawany AA, Fadiel A, Abdel-Hafez MAM. Reproductive performance traits as affected by heat stress and its alleviation in sheep. Tropical Subtropical Agroecosyst. 2008;8 https://doi.org/.

[80] Fowler DG. Semen quality of Merino rams. 1. The effects of fleece length and season on semen quality. Aust J Exp Agr. 1965;5 10.1071/EA9650243.

[81] Kastelic JP, Wilde RE, Rizzoto G, Thundathil JC. Hyperthermia and not hypoxia may reduce sperm motility and morphology following testicular hyperthermia. Veterinární Medicína. 2017;62 10.17221/124/2016-VETMED.

[82] Smith J. The effect of temperature on characteristics of semen of rams. Aust J Agricultural Res. 1971;22 10.1071/AR9710481.

[83] Howarth B. Fertility in the ram following exposure to elevated ambient temperature and humidity. Reproduction. 1969;19 10.1530/jrf.0.0190179. 10.1530/jrf.0.0190179 5793909

[84] Rathore AK. Mid-piece sperm abnormality due to high temperature exposure of rams. British Veterinary Journal. 1969;125 10.1016/S0007-1935(17)48713-6. 10.1016/s0007-1935(17)48713-6 5385350

[85] Rathore AK. Morphological changes in ram spermatozoa due to hot-room exposure for varying periods. British Veterinary Journal. 1970a;126 10.1016/S0007-1935(17)48337-0. 10.1016/s0007-1935(17)48337-0 5472212

[86] Rathore AK. Acrosomal abnormality in ram spermatozoa due to heat stress. Brit Veterinary J. 1970b;126 10.1016/S0007-1935(17)48252-2. 10.1016/s0007-1935(17)48252-2 5485197

[87] Mieusset R, Quintana Casares P, Sanchez Partida LG, Sowerbutts SF, Zupp JL, Setchell BP. Effects of heating the testes and epididymides of rams by scrotal insulation on fertility and embryonic mortality in ewes inseminated with frozen semen. J Reprod Fertility. 1992;94 10.1530/jrf.0.0940337. 10.1530/jrf.0.0940337 1593536

[88] Fowler DG, Dun R. Skin folds and Merino breeding. 4. The susceptibility of rams selected for a high degree of skin wrinkle to heat induced infertility. Aust J Exp Agr. 1966;6 10.1071/EA9660121.

[89] Braden A, Mattner P. The effects of scrotal heating in the ram on semen characteristics, fecundity, and embryonic mortality. Australian Journal of Agricultural Research. 1970;21 10.1071/AR9700509.

[90] Fowler DG, Setchell B. Selecting Merino rams for ability to withstand infertility caused by heat. 2. The effect of heat on scrotal and testicular blood flow. Aust J Exp Agr. 1971;11 10.1071/EA9710143.

[91] Fowler DG. Skin folds and Merino breeding. 7. The relations of heat applied to the testis and scrotal thermoregulation to fertility in the Merino ram. Aust J Exp Agr. 1968;8 10.1071/EA9680142.

[92] Rathore AK. Fertility of rams heated for 1, 2, 3, and 4 days, mated to superovulated ewes. Aust J Agricultural Res. 1970c;21 10.1071/AR9700355.

[93] Rathore AK, editor Effects of high temperature on sperm morphology and subsequent fertility in Merino sheep. Proceeding of the Australian Society for Animal Production; 1968; Armidale: Australian Society for Animal Production.

[94] Zhu B-K, Walker SK, Oakey H, Setchell BP, Maddocks S. Effect of paternal heat stress on the development *in vitro* of preimplantation embryos in the mouse. Andrologia. 2004;36 10.1111/j.1439-0272.2004.00635.x. 10.1111/j.1439-0272.2004.00635.x 15541055

[95] Zhu B-K, Setchell BP. Effects of paternal heat stress on the *in vivo* development of preimplantation embryos in the mouse. Reprod Nutrit Dev. 2004;44/ 10.1051/rnd:2004064. 10.1051/rnd:2004064 15762306

[96] Paul C, Murray AA, Spears N, Saunders PTK. A single, mild, transient scrotal heat stress causes DNA damage, subfertility and impairs formation of blastocysts in mice. Reproduction. 2008;136 10.1530/REP-08-0036. 10.1530/REP-08-0036 18390691

[97] Setchell BP (1978). The mammalian testis.

[98] Maloney SK, Mitchell D. Regulation of ram scrotal temperature during heat exposure, cold exposure, fever and exercise. J Physiol. 1996;496 10.1113/jphysiol.1996.sp021695. 10.1113/jphysiol.1996.sp021695 PMC1160887 8910226

[99] Waites GMH. Polypnoea evoked by heating the scrotum of the ram. Nature. 1961;190 10.1038/190172a0. 10.1038/190172a0 13782573

[100] Waites GMH. Effect of heating scrotum of ram on respiration and body temperature. Quarterly J Experimental Physiol Cognate Med Sci. 1962;47 10.1113/expphysiol.1962.sp001615. 10.1113/expphysiol.1962.sp001615 13998338

[101] Maloney SK, Bonomelli JM, Desouza J. Scrotal heating stimulates panting and reduces body temperature similarly in febrile and non-febrile rams (*Ovis aries*). Comparative Biochem Physiol Part A: Mole Integrative Physiol. 2003;135 10.1016/S1095-6433(03)00139-9. 10.1016/s1095-6433(03)00139-9 12890546

[102] Waites GMH, Voglmayr J. Functional activity and control of apocrine sweat glands of scrotum of ram. Aust J Agricultural Res. 1963;14 10.1071/AR9630839.

[103] Gibson A, Akinrinsola A, Patel T, Ray A, Tucker J, McFadzean I. Pharmacology and thermosensitivity of the dartos muscle isolated from rat scrotum. Brit J Pharmacol. 2002;136 10.1038/sj.bjp.0704830. 10.1038/sj.bjp.0704830 PMC1573456 12163353

[104] Fowler DG, Kennedy J. Skin folds and Merino breeding. 6. The effects of varying heat exposures and degree of skin fold on rectal, scrotal and testis temperatures. Aust J Exp Agr. 1968;8 10.1071/EA9680133.

[105] Moule GR, Waites GM. Seminal degeneration in ram and its relation to temperature of scrotum. J Reprod Fertility. 1963;5 10.1530/jrf.0.0050433. 10.1530/jrf.0.0050433 13936300

[106] Wallage A, Johnston S, Lisle A, Beard L, Lees A, Collins C, et al. Thermoregulation of the bovine scrotum: 1. measurements of free-range animals in a paddock and pen. Int J Biometeorol. 2017;61 10.1007/s00484-017-1315-3. 10.1007/s00484-017-1315-3 28280936

[107] Fowler DG, Waites G. Selecting Merino rams for ability to withstand infertility caused by heat. 1. Anatomy and functional activity of the scrotum. Aust J Exp Agr. 1971;11 10.1071/EA9710137.

[108] Brito LFC, Silva AEDF, Barbosa RT, Kastelic JP. Testicular thermoregulation in *Bos indicus*, crossbred and *Bos taurus* bulls: relationship with scrotal, testicular vascular cone and testicular morphology, and effects on semen quality and sperm production. Theriogenology. 2004;61 10.1016/s0093-691x(03)00231-0. 10.1016/s0093-691x(03)00231-0 14662148

[109] Lindsay DR. Sexual activity and semen production of rams at high temperatures. Reproduction. 1969;18 10.1530/jrf.0.0180001. 10.1530/jrf.0.0180001 5791679

[110] Waites GMH, Ortavant R, editors. Early cytological changes observed after testis was subjected to elevated temperature. Annales de Biologie Animale, Biochimie, Biophysique; 1968: Inst natl recherche agronomique 147 rue de l universite, 75338 paris cedex.

[111] McDonald RM, Smith JF, Montgomery GW, Fleming JS, Cox NR, editors. Sperm DNA damage after scrotal insulation in rams. Proceedings of the New Zealand Society of Animal Production; 2007: New Zealand Society of Animal Production; 1999.

[112] Pérez-Crespo M, Pintado B, Gutiérrez-Adán A. Scrotal heat stress effects on sperm viability, sperm DNA integrity, and the offspring sex ratio in mice. Mole Reprod Dev. 2008;75 10.1002/mrd.20759. 10.1002/mrd.20759 17474098

[113] Houston BJ, Nixon B, Martin JH, De Iuliis GN, Trigg NA, Bromfield EG, et al. Heat exposure induces oxidative stress and DNA damage in the male germ line. Biol Reprod. 2018;98 10.1093/biolre/ioy009. 10.1093/biolre/ioy009 29351587

[114] Ortavant R, Courot M, Hochereau MT. Spermatogenesis and morphology of the spermatozoon. Reproduction in domestic animals: Elsevier; 1969. p. 251–276.

[115] Rizzoto G, Hall C, Tyberg JV, Thundathil JC, Caulkett NA, Kastelic JP. Testicular hyperthermia increases blood flow that maintains aerobic metabolism in rams. Reprod Fertility Dev. 2018;31 10.1071/RD17509. 10.1071/RD17509 30449297

[116] Aitken RJ, Gibb Z, Baker MA, Drevet J, Gharagozloo P. Causes and consequences of oxidative stress in spermatozoa. Reprod Fertility Dev. 2016;28 10.1071/RD15325. 10.1071/RD15325 27062870

[117] Paul C, Teng S, Saunders PTK. Single, mild, transient scrotal heat stress causes hypoxia and oxidative stress in mouse testes, which induces germ cell death. Biol Reprod. 2009;80 10.1095/biolreprod.108.071779. 10.1095/biolreprod.108.071779 PMC2709966 19144962

[118] Guan Y, Martin GB. Cellular and molecular responses of adult testis to changes in nutrition: novel insights from the sheep model. Reproduction. 2017;154 10.1530/REP-17-0061. 10.1530/REP-17-0061 28982938

[119] Maurya VP, Sejian V, Kumar D, Naqvi SMK. Impact of heat stress, nutritional restriction and combined stresses (heat and nutritional) on growth and reproductive performance of Malpura rams under semi-arid tropical environment. J Anim Physiol Animal Nutrit. 2016;100 10.1111/jpn.12443. 10.1111/jpn.12443 26718122

[120] Galan HL, Hussey MJ, Barbera A, Ferrazzi E, Chung M, Hobbins JC, et al. Relationship of fetal growth to duration of heat stress in an ovine model of placental insufficiency. Am J Obstetrics and Gynecol. 1999;180 10.1016/S0002-9378(99)70629-0. 10.1016/s0002-9378(99)70629-0 10329890

[121] Barbera A, Jones III OW, Zerbe GO, Hobbins JC, Battaglia FC, Meschia G. Early ultrasonographic detection of fetal growth retardation in an ovine model of placental insufficiency. Am J Obstetrics Gynecol. 1995;173 10.1016/0002-9378(95)91328-9. 10.1016/0002-9378(95)91328-9 7485295

[122] Regnault TR, Orbus RJ, Battaglia FC, Wilkening RB, Anthony RV. Altered arterial concentrations of placental hormones during maximal placental growth in a model of placental insufficiency. J Endocrinol. 1999;162 10.1677/joe.0.1620433. 10.1677/joe.0.1620433 10467235

[123] Regnault TRH, Orbus RJ, De Vrijer B, Davidsen ML, Galan HL, Wilkening RB, et al. Placental expression of VEGF, PlGF and their receptors in a model of placental insufficiency—intrauterine growth restriction (PI-IUGR). Placenta. 2002;23 10.1053/plac.2001.0757. 10.1053/plac.2001.0757 11945079

[124] De Vrijer B, Davidsen ML, Wilkening RB, Anthony RV, Regnault TRH. Altered placental and fetal expression of IGFs and IGF-binding proteins associated with intrauterine growth restriction in fetal sheep during early and mid-pregnancy. Pediatric Research. 2006;60 https://doi.org/articles/pr2006339. 10.1203/01.PDR.0000242364.78002.71 16966353

[125] De Vrijer B, Regnault TRH, Wilkening RB, Meschia G, Battaglia FC. Placental uptake and transport of ACP, a neutral nonmetabolizable amino acid, in an ovine model of fetal growth restriction. Am J Physiol Endocrinol Metabolism. 2004;287 10.1152/ajpendo.00259.2004. 10.1152/ajpendo.00259.2004 15315907

[126] Thureen PJ, Trembler KA, Meschia G, Makowski EL, Wilkening RB. Placental glucose transport in heat-induced fetal growth retardation. Am J Physiol Regulatory Integrative Comparative Physiol. 1992;263 10.1152/ajpregu.1992.263.3.R578. 10.1152/ajpregu.1992.263.3.R578 1415644

[127] Shelton M, Huston JE. Effects of high temperature stress during gestation on certain aspects of reproduction in the ewe. J Anim Sci. 1968;27 10.2527/jas1968.271153x. 10.2527/jas1968.271153x 5637649

[128] Brown DE, Harrison PC, Hinds FC, Lewis JA, Wallace MH. Heat stress effects on fetal development during late gestation in the ewe. J Anim Sci. 1977;44 10.2527/jas1977.443442x. 10.2527/jas1977.443442x 845090

[129] McCrabb GJ, McDonald BJ, Hennoste LM. Heat stress during mid-pregnancy in sheep and the consequences for placental and fetal growth. J Agricultural Sci. 1993;120 10.1017/s0021859600074311.

[130] Bell AW, Wilkening RB, Meschia G. Some aspects of placental function in chronically heat-stressed ewes. J Dev Physiol. 1987;9 https://doi.org/abstract/med/3559063. 3559063

[131] Early RJ, McBride BW, Vatnick I, Bell AW. Chronic heat stress and prenatal development in sheep: 2. Placental cellularity and metabolism. J Anim Sci. 1991;69 10.2527/1991.6993610x. 10.2527/1991.6993610x 1718933

[132] Bell AW, McBride BW, Slepetis R, Early RJ, Currie WB. Chronic heat stress and prenatal development in sheep: 1. Conceptus growth and maternal plasma hormones and metabolites. J Anim Sci. 1989;67 10.2527/jas1989.67123289x. 10.2527/jas1989.67123289x 2613577

[133] Alexander G, Williams D. Heat stress and development of the conceptus in domestic sheep. J Agricultural Sci. 1971;76 10.1017/S0021859600015616.

[134] Cartwright GA, Thwaites CJ. Foetal stunting in sheep: 1. The influence of maternal nutrition and high ambient temperature on the growth and proportions of Merino foetuses. J Agricultural Sci. 1976;86 10.1017/S0021859600061128.

[135] Yeates NTM. The effect of high air temperature on reproduction in the ewe. J Agricultural Sci. 1953;43 10.1017/S002185960004497X.

[136] Yeates NTM. Foetal dwarfism in sheep—an effect of high atmospheric temperature during gestation. J Agricultural Sci. 1958;51 10.1017/S0021859600032846.

[137] Yapi CV, Boylan WJ, Robinson RA. Factors associated with causes of preweaning lamb mortality. Preventive Veterinary Med. 1990;10 10.1016/0167-5877(90)90060-U.

[138] Fogarty NM, Hopkins DL, van de Ven R. Lamb production from diverse genotypes. 1. Lamb growth and survival and ewe performance. Anim Sci J. 2000;70 10.1017/S1357729800051675.

[139] Geenty KG, Brien FD, Hinch GN, Dobos RC, Refshauge G, McCaskill M, et al. Reproductive performance in the Sheep CRC Information Nucleus using artificial insemination across different sheep-production environments in southern Australia. Anim Prod Sci. 2014;54 10.1071/AN11323.

[140] Scales GH, Burton RN, Moss RA. Lamb mortality, birthweight, and nutrition in late pregnancy. New Zealand J Agricultural Res. 1986;29 10.1080/00288233.1986.10417977.

[141] Alexander G, Williams D. Shivering and non-shivering thermogenesis during summit metabolism in young lambs. J Physiol. 1968;198 10.1113/jphysiol.1968.sp008605. 10.1113/jphysiol.1968.sp008605 PMC1365322 5698273

[142] Regnault TRH, de Vrijer B, Galan HL, Davidsen ML, Trembler KA, Battaglia FC, et al. The relationship between transplacental O2 diffusion and placental expression of PlGF, VEGF and their receptors in a placental insufficiency model of fetal growth restriction. J Physiol. 2003;550 10.1113/jphysiol.2003.039511. 10.1113/jphysiol.2003.039511 PMC2343042 12740423

[143] Galan HL, Hussey MJ, Chung M, Chyu JK, Hobbins JC, Battaglia FC. Doppler velocimetry of growth-restricted fetuses in an ovine model of placental insufficiency. American Journal of Obstetrics and Gynecology. 1998;178 10.1016/S0002-9378(98)70419-3. 10.1016/s0002-9378(98)70419-3 9539507

[144] Symonds ME, Sebert SP, Budge H. Nutritional regulation of fetal growth and implications for productive life in ruminants. Animal. 2010;4 10.1017/S1751731110000479. 10.1017/S1751731110000479 22444610

[145] Herring CM, Bazer FW, Johnson GA, Wu G. Impacts of maternal dietary protein intake on fetal survival, growth, and development. Experim Biol Med. 2018;243 10.1177/1535370218758275. 10.1177/1535370218758275 PMC5882021 29466875

[146] Vonnahme KA, Lemley CO, Shukla P, Oerourke ST. Placental programming: how the maternal environment can impact placental function. J Anim Sci. 2013;91 10.2527/jas.2012-5929. 10.2527/jas.2012-5929 23307854

[147] Brown DE, Harrison PC. Central sympathetic control of uterine blood flow during acute heat stress. J Anim Sci. 1981;52 10.2527/jas1981.5251114x. 10.2527/jas1981.5251114x 7240051

[148] Vatnick I, Ignotz G, McBride BW, Bell AW. Effect of heat stress on ovine placental growth in early pregnancy. J Dev Physiol. 1991;16 https://doi.org/abstract/med/1797923. 1797923

[149] Gilbert RD, Schroder H, Kawamura T, Dale PS, Power GG. Heat transfer pathways between fetal lamb and ewe. J Appl Physiol. 1985;59 10.1152/jappl.1985.59.2.634. 10.1152/jappl.1985.59.2.634 4030617

[150] Oakes GK, Walker AM, Ehrenkranz RA, Cefalo RC, Chez RA. Uteroplacental blood flow during hyperthermia with and without respiratory alkalosis. J Applied Physiol. 1976;41 10.1152/jappl.1976.41.2.197. 10.1152/jappl.1976.41.2.197 956102

[151] Laburn H, Faurie A, Goelst K, Mitchell D. Effects on fetal and maternal body temperatures of exposure of pregnant ewes to heat, cold, and exercise. J Appl Physiol. 2002;92 10.1152/japplphysiol.00109.2001. 10.1152/japplphysiol.00109.2001 11796695

[152] Laburn HP, Mitchell D, Goelst K. Fetal and maternal body temperatures measured by radiotelemetry in near-term sheep during thermal stress. J Appl Physiol. 1992;72 10.1152/jappl.1992.72.3.894. 10.1152/jappl.1992.72.3.894 1568984

[153] Andrianakis P, Walker D. Effect of hyperthermia on uterine and umbilical blood flows in pregnant sheep. Experim Physiol. 1994;79 10.1113/expphysiol.1994.sp003735. 10.1113/expphysiol.1994.sp003735 8011310

[154] Hopkins PS, Nolan CJ, Pepper P. The effects of heat stress on the development of the foetal lamb. Aust J Agricultural Res. 1980;31 10.1071/AR9800763.

[155] McCrabb GJ, Bortolussi G. Placental growth and the ability of sheep to thermoregulate in hot environment. Small Ruminant Res. 1996;20 10.1016/0921-4488(95)00802-0.

[156] Tao S, Dahl G, Laporta J, Bernard J. Effects of heat stress during late gestation on the dam and its calf. J Anim Sci. 2018;96 10.1093/jas/skz061. 10.1093/jas/skz061 PMC6488308 30753515

[157] Rhoads ML. Effects of periconceptional heat stress on primiparous and multiparous daughters of holstein dairy cows. Theriogenology. 2020;In Press 10.1016/j.theriogenology.2020.03.015. 10.1016/j.theriogenology.2020.03.015 32234245

[158] Ouellet V, Laporta J, Dahl GE. Late gestation heat stress in dairy cows: effects on dam and daughter. Theriogenology. 2020;In Press 10.1016/j.theriogenology.2020.03.011. 10.1016/j.theriogenology.2020.03.011 32278591

[159] Tao S, Monteiro APA, Thompson IM, Hayen MJ, Dahl GE. Effect of late-gestation maternal heat stress on growth and immune function of dairy calves. J Dairy Sci. 2012;95 https://doi.org/. 10.3168/jds.2012-5697 23021751

[160] Monteiro APA, Tao S, Thompson IMT, Dahl GE. *In utero* heat stress decreases calf survival and performance through the first lactation. J Dairy Sci. 2016;99 10.3168/jds.2016-11072. 10.3168/jds.2016-11072 27522427

[161] Skibiel AL, Dado-Senn B, Fabris TF, Dahl GE, Laporta J. *In utero* exposure to thermal stress has long-term effects on mammary gland microstructure and function in dairy cattle. PloS One. 2018;13 10.1371/journal.pone.0206046. 10.1371/journal.pone.0206046 PMC6191142 30325972

[162] Ahmed BMS, Younas U, Asar TO, Dikmen S, Hansen PJ, Dahl GE. Cows exposed to heat stress during fetal life exhibit improved thermal tolerance. J Anim Sci. 2017;95 10.2527/jas.2016.1298. 10.2527/jas.2016.1298 28805919

[163] Johnson JS, Boddicker RL, Sanz-Fernandez MV, Ross JW, Selsby JT, Lucy MC, et al. Effects of mammalian *in utero* heat stress on adolescent body temperature. Int J Hyperthermia. 2013;29 10.3109/02656736.2013.843723. 10.3109/02656736.2013.843723 24102398

[164] Johnson JS, Sanz Fernandez MV, Seibert JT, Ross JW, Lucy MC, Safranski TJ, et al. *In utero* heat stress increases postnatal core body temperature in pigs. J Anim Sci. 2015;93 10.2527/jas.2015-9112. 10.2527/jas.2015-9112 26440331

[165] Johnson JS, Baumgard LH. Physiology symposium: postnatal consequences of *in utero* heat stress in pigs. J Anim Sci. 2019;97 10.1093/jas/sky472. 10.1093/jas/sky472 PMC6358235 30534960

[166] Dado-Senn B, Skibiel AL, Fabris TF, Dahl GE, Laporta J. Dry period heat stress induces microstructural changes in the lactating mammary gland. PloS One. 2019;14 10.1371/journal.pone.0222120. 10.1371/journal.pone.0222120 PMC6752841 31536517

[167] Dahl GE, Tao S, Laporta J. TRIENNIAL LACTATION SYMPOSIUM/BOLFA: Late gestation heat stress of dairy cattle programs dam and daughter milk production. J Anim Sci. 2017;95 10.2527/jas2017.2006. 10.2527/jas2017.2006 PMC6292301 29293764

[168] Akers RM. Lactogenic hormones: binding sites, mammary growth, secretory cell differentiation, and milk biosynthesis in ruminants. J Dairy Sci. 1985;68 10.3168/jds. S0022-0302(85)80849–3. 10.3168/jds.s0022-0302(85)80849-3 3886733

[169] Swanson T, Hammer C, Luther J, Carlson D, Taylor J, Redmer D, et al. Effects of gestational plane of nutrition and selenium supplementation on mammary development and colostrum quality in pregnant ewe lambs1. J Anim Sci. 2008;86 10.2527/jas.2008-0996. 10.2527/jas.2008-0996 18441080

[170] Tygesen MP, Nielsen MO, Nørgaard P, Ranvig H, Harrison AP, Tauson A-H. Late gestational nutrient restriction: effects on ewes' metabolic and homeorhetic adaptation, consequences for lamb birth weight and lactation performance. Archives Anim Nutrit. 2008;62 10.1080/17450390701780276. 10.1080/17450390701780276 18341079

[171] Sevi A, Caroprese M. Impact of heat stress on milk production, immunity and udder health in sheep: a critical review. Small Ruminant Res. 2012;107 10.1016/j.smallrumres.2012.07.012.

[172] Ramón M, Díaz C, Pérez-Guzman MD, Carabaño MJ. Effect of exposure to adverse climatic conditions on production in Manchega dairy sheep. J Dairy Sci. 2016;99 10.3168/jds.2016-10909. 10.3168/jds.2016-10909 27132106

[173] Contreras-Jodar A, Salama AAK, Hamzaoui S, Vailati-Riboni M, Caja G, Loor JJ. Effects of chronic heat stress on lactational performance and the transcriptomic profile of blood cells in lactating dairy goats. J Dairy Res. 2018;85 10.1017/S0022029918000705. 10.1017/S0022029918000705 30236165

[174] Smith ID, Bell GH, De Chaneet G. Embryonic mortality in Merino ewes exposed to high ambient temperatures. Aust Vet J. 1966;42 10.1111/j.1751-0813.1966.tb14478.x. 10.1111/j.1751-0813.1966.tb14478.x 5962490

[175] Sejian V, Maurya VP, Kumar K, Naqvi SMK. Effect of multiple stresses (thermal, nutritional, and walking stress) on the reproductive performance of Malpura ewes. Veterinary Med Int. 2012;2012 10.1155/2012/471760. 10.1155/2012/471760 PMC3289860 22448337

[176] Spies HG, Menzies CS, Scott SP, Coon LL, Kiracofe GH. Effects of forced exercise and cooling on reproductive performance of finewool ewes bred during the summer. J Anim Sci. 1965;24 10.2527/jas1965.2419. 10.2527/jas1965.2419 14296201

[177] Sejian V, Maurya VP, Naqvi SMK. Effect of thermal stress, restricted feeding and combined stresses (thermal stress and restricted feeding) on growth and plasma reproductive hormone levels of Malpura ewes under semi-arid tropical environment. J Anim Physiol Anim Nutrit. 2011;95 10.1111/j.1439-0396.2010.01048.x. 10.1111/j.1439-0396.2010.01048.x 20796074

[178] Kumar D, De K, Shekhawat I, Bahadur S, Balaganur K, Naqvi SMK. Combined effect of heat and nutritional stress on superovulation of Malpura ewes in a semi-arid region. J Thermal Biol. 2019;80 10.1016/j.jtherbio.2019.02.007. 10.1016/j.jtherbio.2019.02.007 30784480

